# Correlation of LAGE3 with unfavorable prognosis and promoting tumor development in HCC via PI3K/AKT/mTOR and Ras/RAF/MAPK pathways

**DOI:** 10.1186/s12885-022-09398-3

**Published:** 2022-03-21

**Authors:** Yun Li, Hui Xiong

**Affiliations:** Department of General Surgery, the First Affiliated Hospital of Nanchang University, Nanchang, 330000 China

**Keywords:** HCC, LAGE3, Proliferation, Migration, Invasion, Apoptosis

## Abstract

**Background:**

Hepatocellular carcinoma (HCC) is one of the most common clinical malignancies quite susceptible to recurrence and metastasis. Despite several improvements in therapeutic approaches, the prognosis remains poor due to the limited treatment options. A bioinformatics analysis based on TCGA databases revealed that the recombinant human L antigen family member 3 (LAGE3) might function as an effective prognostic and diagnostic biomarker for HCC, as LAGE3, a protein-coding gene, maintains several important biological functions and has a physiological significance in the CTAG family while simultaneously being involved in regulating the occurrence and invasion of numerous types of tumors. However, the LAGE3 gene’s functional and regulatory mechanism in the progression of HCC remains unclear.

**Methods:**

The LAGE3 level was investigated in 79 HCC tissues cases, ten HCC adjacent tissue cases, and six cases of normal liver tissues by IHC, while the LAGE3 level was evaluated in BEL-7404, SMCC-7721, Huh-7, HepG2, and MIHA cell lines by qRT-PCR and Western blot tests. Although the proliferation, migration, invasion, and apoptotic abilities of HCC cells were measured in vitro after silencing assay to probe the role of LAGE3 in HCC cells, the tumor xenograft growth experiment was used to verify the in vivo effect of LAGE3 gene knockdown on the growth of HCC tumors combined with bioinformatics analysis to study the LAGE3 mechanisms regulating HCC proliferation.

**Results:**

Our results implied that LAGE3 was extensively expressed in HCC cell lines like BEL-7404, SMCC-7721, and Huh-7 cells as well as HCC tissues, but a lower expression was observed in HepG2 cells. Additionally, LAGE3 restrains cellular proliferation, promotes apoptotic pathways in HCC cells, and inhibits the growth of HCC tumors in vivo. Lastly, it was stated that LAGE3 might promote tumor development in HCC via PI3K/AKT/mTOR and Ras/RAF/MAPK pathways.

**Conclusion:**

This study shows that the development of specific LAGE3 target drugs might become new effective treatment modalities for HCC patients.

**Supplementary Information:**

The online version contains supplementary material available at 10.1186/s12885-022-09398-3.

## Introduction

Hepatocellular carcinoma (HCC) is one of the most common clinical malignancies and a leading cause of mortality worldwide. Despite all the recent technological advancements, HCC is quite susceptible to recurrence and metastasis, while the treatment options for the same are limited, and hence, prognosis remains poor [1]. In the present scenario, several promising therapeutic drugs for treating HCC have been vastly improvised due to the discovery of few novel targets related to several biological signaling pathways, like cell epithelial-mesenchymal transition (EMT) that plays a crucial role in the early steps of the metastatic process and tyrosine kinase activation in HCC [2, 3]. Since various disease-directed treatment options for HCC are still very sparse, it is imperative to seek more specific and effective HCC therapeutic targets.

Recombinant human L antigen family member 3 (LAGE3) is a part of the CTAG family ubiquitously expressed in many cell types and is often considered a notable up-regulated RNA modification-related protein in a majority of carcinoma cases [4]. Previous studies have consistently observed that an extensive expression of LAGE3 is associated with poor prognosis and poor immune infiltration in patients with papillary thyroid carcinoma (PTC) and cutaneous melanoma (CM) [5–8]. Dong X et al. found that LAGE3 enhanced the abilities of proliferation, migration, and invasion of cancer cells and resisted their apoptosis by activating intrinsic intracellular signaling pathways, thereby facilitating the development of breast cancer (BC) [9]. A study by Goswami T et al. displayed that knockdown of LAGE3 affected cellular proliferation, thereby significantly reducing the growth of non-small cell lung cancer cells [10]. Zhang Y et al. discovered that lncRNA NEAT1 facilitated cellular proliferation and migration via serving as a miR-320a molecular sponge and targeting LAGE3 in liver cancer [11]. However, the clinical significance of LAGE3 has not been thoroughly elucidated in the carcinogenesis and development of HCC.

RAS/RAF/MAPK and PI3K/AKT/mTOR signal pathways, which involve cell proliferation, survival, invasion, migration, apoptosis, glucose metabolism, and DNA repair, are up-regulated oncogenic cascade signals in a variety of tumors [12–14]. For example, PI3K/AKT/mTOR signal up-regulation stimulates breast cancer progression, and PI3K/AKT/mTOR axis inhibitors have shown positive significance in breast cancer treatment. The abnormal activation of Ras-Raf-MAPK signal promotes the proliferation of gastric cancer cells. Further, phosphorylation of the Ras/Raf/MAPK and PI3K/Akt pathways enhances the resistance of sorafenib in hepatocellular carcinoma. Whether the abnormal activation of the above two pathways is involved in LAGE3 in promoting liver cancer progression is still unknown.

Although our study evaluated the LAGE3 levels in various HCC cells and tissues based on TCGA and GEO public datasets by employing bioinformatics, the obtained results indicated that an extensive expression of LAGE3 was associated with poor prognosis in HCC patients. An additional investigation into the biological function and latent pathways related to LAGE3 revealed that LAGE3 might promote tumor development in HCC via intrinsic PI3K/AKT/mTOR and Ras/RAF/MAPK pathways, the development of specific LAGE3 target drugs might become novel treatment strategies for HCC patients.

## Materials and methods

### Survival analysis and gene expression analysis

The “Expression analysis-Box Plots” module of the Gene expression profiling interactive analysis, Version 2 (GEPIA2) webserver (http://gepia2.cancer-pku.cn/#analysis) was employed to obtain box plots of the differences in expression between the liver hepatocellular carcinoma (LIHC) tissues and the corresponding normal tissues, with a *p*-value cutoff of 0.01 and log_2_FC (Fold change) cutoff of 1. Additionally, the “Survival Analysis” module of GEPIA2 was also utilized to obtain the survival plots of the OS (Overall survival) and DFS (Disease-free survival). Cutoff-high (50%) and cutoff-low (50%) values were selected as the expression thresholds for splitting the high-expression and low-expression cohorts while using the log-rank test as the hypothesis test.

### Cell lines and cell culture

BEL-7404, SMCC-7721, Huh-7, HepG2, and MIHA cells were brought from the Shanghai Cell Bank Collection (Shanghai, China) and cultured in Dulbecco’s Modified Eagle’s Medium (DMEM, Corning, Tauranga, New Zealand) supplemented with 10% fetal bovine serum (Hangzhou Sijiqing Biological Engineering Materials, China) and maintained in an incubator at 37 °C, 5% CO_2_.

### Quantitative real-time PCR

Total cellular RNA was collected by TRIzol® Plus RNA Purification Kit (Invitrogen, #12183–555) and RNase-Free DNase Set (Qiagen, #79254) reagents according to the provided lysate preparation protocol. The cDNA was synthesized using SuperScript™ III First-Strand Synthesis SuperMix (Invitrogen, #11752–050) while the PCR was performed using Power SYBR® Green PCR Master Mix (Applied Biosystems, #4367659) following the reaction instructions and conditions of the manufacturer’s protocol. The primers were designed by Primer Premier 6.0 and Beacon designer 7.8 software and are listed in Table 1.

**Table 1:** Real-time PCR primers and conditions

Gene	GenBank Accession	Primer Sequences(5’to3’)	Size (bp)	Annealing (°C)
GAPDH	NM_002046.5	CCATGACAACTTTGGTATCGTGGAA GGCCATCACGCCACAGTTTC	107	60
LAGE3	NM_006014.4	GGCCGCACATATTCACCCTC TCTTCAGCTTTCCAGCGGAC	160	60
AKT	NM_001014431.2	CGAGCTGTTCTTCCACCTGT CGACCGCACATCATCTCGTA	339	60
BRAF	NM_001354609.2	TGTTCAACGGGGACATGGAG CACCTTGCAGGTACCACTGT	466	60
ERK	NM_002746	ATGTCATCGGCATCCGAGAC GGATCTGGTAGAGGAAGTAGCA	156	60
PI3CA	NM_006218.4	GAGGTTTGGCCTGCTTTTGG TCCCACACAGTCACCGATTG	493	60
MAP2K1	NM_002755.4	ATGCCCAAGAAGAAGCCGAC AGCTCTAGCTCCTCCAGCTT	125	60
KRAS	NM_001369786.1	CAGTGCAATGAGGGACCAGT AGCATCCTCCACTCTCTGTCT	274	60

### Immunohistochemistry (IHC)

After dewaxing, the tissue chip (#D950601, Shanghai Baioujing Biological Technology Co., Ltd.) underwent a heat-induced citric acid antigen retrieval method and was then cooled to room temperature. Since the endogenous peroxidase activity was blocked for 15 mins at room temperature by adding 3% H_2_O_2_, the tissue chip was incubated with the primary LAGE3 antibody (1: 500, Biorbyt, #orb327020) and then with secondary antibody at 37 °C for 1 h. Lastly, DAB chromogen was used to generate a colored precipitate, after which the color endpoint was determined under a microscope. Finally, the nuclei were counterstained with hematoxylin for 2 mins. The resultant colors of both cell nuclei as blue and the positive expression site as brown-yellow were used as the standard IHC criterion.

### Western blot

The total protein was extracted and quantified by BCA Protein Assay Kit (Beyotime, #P0010). After a required amount of 60 μg total protein was loaded per well and electrophoresed, the PVDF membrane (Millipore, #IPVH00010) was blocked in 5% bovine serum albumin (BSA, Sangon Biotech, #A600903). The primary antibodies were incubated overnight at 4 °C, followed by incubating secondary antibodies at room temperature for 1 h. Subsequently, ECL DualVue WB Marker (GE, #RPN810) was used to prepare a 1 mL ECL working development solution. The information on various utilized antibodies is mentioned in Tables 2 and 3.

**Table 2:** The information of primary antibodies in Western Blotting

Name of Antibodies	Brand and Item Number	Dilution	Molecular weight (kDa)
LAGE3	Biorbyt orb327020	1: 500	14
Ras	CST 3339	1: 1000	21
Raf	Abcam ab137435	1:3000	73
PI3K	Abcam ab86714	1:1000	85
P-Akt (Ser473)	CST 4060	1: 1000	60
Akt (Pan)	CST 4691	1: 1000	60
P-MEK1	CST 9127	1:1000	45
MEK1 (p-S298)	CST 2352	1:1000	45
p-Erk	CST 4376	1: 1000	42, 44
Erk (MAPK1)	CST 4695	1: 1000	42, 44
Braf	Abcam ab33899	1: 2000	90
GAPDH	Abcam ab181602	1: 10000	36

**Table 3:** The information of secondary antibodies in Western Blotting

Name of Antibodies	Brand and Item Number	Dilution
Goat anti-Mouse IgG(H + L) Secondary antibody	Thermo Pierce, #31160	1:5000
Goat anti-Rabbit IgG(H + L) Secondary antibody	Thermo Pierce, #31210	1:5000

### LAGE3-siRNA mediated gene silencing

After reaching 90% confluence, the cells were transfected with 0.5 mM LAGE3-specific siRNA lentiviral particles or control siRNA lentivirus particles (RUANTUO, Shanghai, China). The siRNA target sequences of LAGE3 were.

siLAGE3–1: 5′-GCUCCGAAUUUCCGUCAUCAATT-3′ (forward).

and 5′-UUGAUGACGGAAAUUCGGAGCTT-3′ (reverse),

siLAGE3–2: 5′-CAUCAACUUUCUUGACCAGCUTT-3′ (forward).

and 5′-AGCUGGUCAAGAAAGUUGAUGTT-3′ (reverse),

siLAGE3–3: 5′-GCGCUCUGGAAUCGAGUUATT-3′ (forward).

and 5′-UAACUCGAUUCCAGAGCGCTG-3′ (reverse).

### BRAF overexpression

After reaching 90% confluence, the cells were transfected with 0.5 mM LAGE3 gene lentiviral particles or empty plasmid lentiviral particles (RUANTUO, Shanghai, China). Primers for gene overexpression are mentioned in Supplementary materials.

### Scratch assay

The cells were seeded in a 12-well plate with a seeding density of 1.2 × 10^6^/mL with two uniform horizontal lines on the back. When the cells reached 90% confluence, the wells were scratched along a ruler with a sterile 10 μL pipette tip, perpendicular to the horizontal lines on the back, and rinsed gently with PBS three times. Then, the wells were supplemented with a culture medium containing 0.5% FBS and cultured in a 37 °C, 5% CO_2_ incubator. Lastly, all the images were taken at 24 and 48 h, respectively. The ImageJ plugin software was utilized to quantify the different in vitro parameters like measuring the migration distance of each group of cells after scratching and calculating the migration rate with high accuracy.

### Transwell cell migration assay

According to the reagent’s operating instructions, 100 μL of serum-free cell suspension with a concentration of 3 × 10^5^ cells/mL and 600 μL of 30% FBS culture medium were supplemented to the upper and lower chambers, respectively, and then cultured at 37 °C, 5% CO_2_ in a humidified atmosphere for 48 h. After aspirating the medium from the upper chamber and removing non-transformed cells, the lower surface of the chamber membrane was stained with 1% crystal violet for 15 mins and naturally dried.

### Transwell invasion assay

Transwell upper and lower chambers were supplemented with 500 μL of serum-free medium and placed in a 37 °C, 5% CO_2_ incubator for 2 h to rehydrate the Matrigel matrix layer (BD, #356234), and then were transferred to a new 24-well plate. After that, 100 μL of serum-free cell suspension with a concentration of 3 × 10^5^ cells/mL was added to the upper chamber while 600 μL of 10% FBS culture medium was added to the lower chamber and cultured for 48 h. The next operation was performed in the same way as the scratch assay.

### BrdU assay

One hundred microliter of cell suspension with a density of 1 × 10^4^ cells/mL was added into a 96-well plate. On the first and fourth days after plating, 100 μL of 20 μM EDU working solution (Beyotime, #C0088S) was preheated at 37 °C and added into the 96-well plate followed by incubation for 2 h. The cells were treated with Triton X-100 and then incubated with endogenous peroxidase blocking solution for 20 mins at room temperature. Subsequently, 50 μL of the click reaction solution and the streptavidin-HRP working solution were supplemented in a proper sequence. Finally, the cells were incubated with 0.1 mL TMB color developing a solution for 15 min while the reaction was stopped by using 25 μL 2 M H_2_SO_4_ solution followed by the absorbance measurement at 450 nm.

### CCK8 assay

The cells were seeded in a 96-well plate at a seeding density of 1.0 × 10^3^/mL and were incubated at 37 °C for 24, 48, 72, 96, and 120 h., respectively. Later, the wells were supplemented with 10 μL Cell Counting Kit 8 solutions (CCK8, Biomiky, #BL001B 500 T) and incubated for 4 h, followed by measurement at 450 nm.

### Cell cycle analysis

At 80% confluency, the cells were trypsinized and then resuspended in a complete growth medium. After the cells were collected with a 5 mL centrifuge tube and fixed with 75% ethanol, the cells were stained with 600 μL staining solution for 30 mins.

### Apoptosis analysis

The cells were trypsinized and resuspended in a complete growth medium at 80% confluency in a 6-well plate and collected in the 5 mL centrifuge tube with the supernatant cells and were centrifuged. After the supernatant was discarded and the cells were washed with pre-cooled PBS at 4 °C, subsequently, the cells were stained with 2 μL Annexin V-FITC and PI solution (Beyotime, #C1062L) at room temperature for 30 mins.

### The xenograft assay

All animal experiments were approved by the Experimental Animal Ethics Committee of Nanchang University. The right flanks of nude mice (Shanghai Lingchang Biotechnology Co., Ltd) were subcutaneously injected with a Huh7 cell suspension of 5 × 10^6^ cells/200 μL transfected with siLAGE3 or siCtrl by using disposable sterile syringes. When the tumor volume value reached 5 mm, tumor size and animal weight were measured once every 3 days. After 18 days of subcutaneous injection, the mice were euthanized by an overdose of 2% sodium pentobarbital, and the death was confirmed by cervical dislocation. The removed tumors were weighed and placed in paraformaldehyde to be fixed at room temperature.

### Statistical analysis

All experiments were carried out three times independently and analyzed with Student’s t-test and a two-way ANOVA test. The *p* < 0.05 was considered statistically significant while all analyses were performed using the GraphPad Prism 5. (GraphPad Software, Inc., San Diego, CA).

## Results

### Overexpression of LAGE3 in HCC

**Fig. 1:**
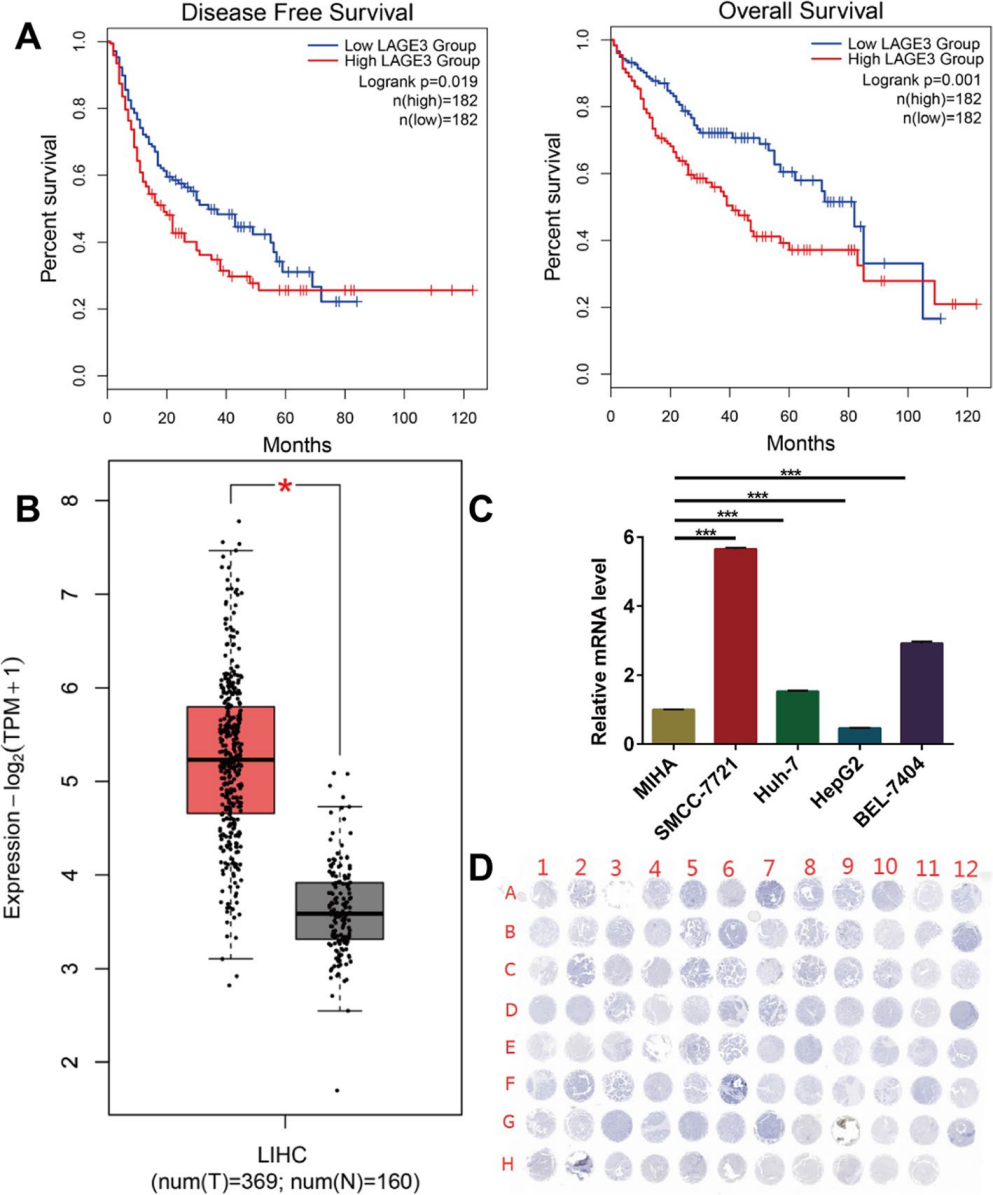
Overexpression of LAGE3 in HCC tissues and cell lines. **A** Usage of GEPIA2 tool to perform OS and DFS analyses of LIHC tumors in TCGA by LAGE3 gene expression for obtaining Kaplan-Meier curves. **B** The LAGE3 expression analyses in LIHC tumors as included in TCGA were performed by the GEPIA2 tool and the box plot data were supplied. * *p* < 0.05. **C** High expression of LAGE3 mRNA level in HCC cell lines while the data were expressed as the mean ± s. d., *** *p* < 0.001. **D** IHC staining images of LAGE3 expressions in HCC tumor tissue chips. OS: Overall survival; DFS: Disease-free survival; LIHC: liver hepatocellular carcinoma; IHC: Immunohistochemistry

We divided the cancer cases into high-expression and low-expression groups according to the expression levels of APC and investigated the correlation between LAGE3 expression and the prognosis of LIHC patients with the help of TCGA datasets. An analysis of the survival data revealed that a LAGE3 overexpression was associated with a poor prognosis of OS (*p* = 0.001) and DFS (Fig. 1A, *p* = 0.019), while the Gene Expression Profiling Interactive Analysis 2 (GEPIA) database tool also confirmed that LIHC tumor tissues highly expressed LAGE3. (Fig. 1B, *p* < 0.05). The above data indicated that APC expression is closely associated with the prognosis of cases with LIHC. Additionally, the LAGE3 expression detected by qRT-PCR in vitro indicated that LAGE3 was highly expressed in SMCC-7721, Huh-7, and BEL-7404 cell lines (*p* < 0.001), whereas a low expression was observed in the HepG2 cell line. (Fig. 1C, *p* < 0.001)). Henceforth, SMCC-7721 and Huh-7 cell lines were chosen to evaluate LAGE3 in LIHC patients further. Moreover, the IHC investigation of the LAGE3 expression in HCC tissue chips demonstrated that LAGE3 expression was relatively higher in 79 HCC cases (A1-A12, B1-B12, C1-C12, D1-D12, E1-E12, F1-F12, and G1-G7) when compared to ten cases of adjacent non-tumor liver tissue (G8-G12 and H1-H5) and six cases of normal liver tissue (H6-H11) (Fig. 1D). The above results collectively indicated that LAGE3 might be an important molecular target to promote tumor progression in HCC.

### Silencing LAGE3 by infection of lentivirus-mediated siRNA to explore the effect of LAGE3 suppression on HCC progression

**Fig. 2:**
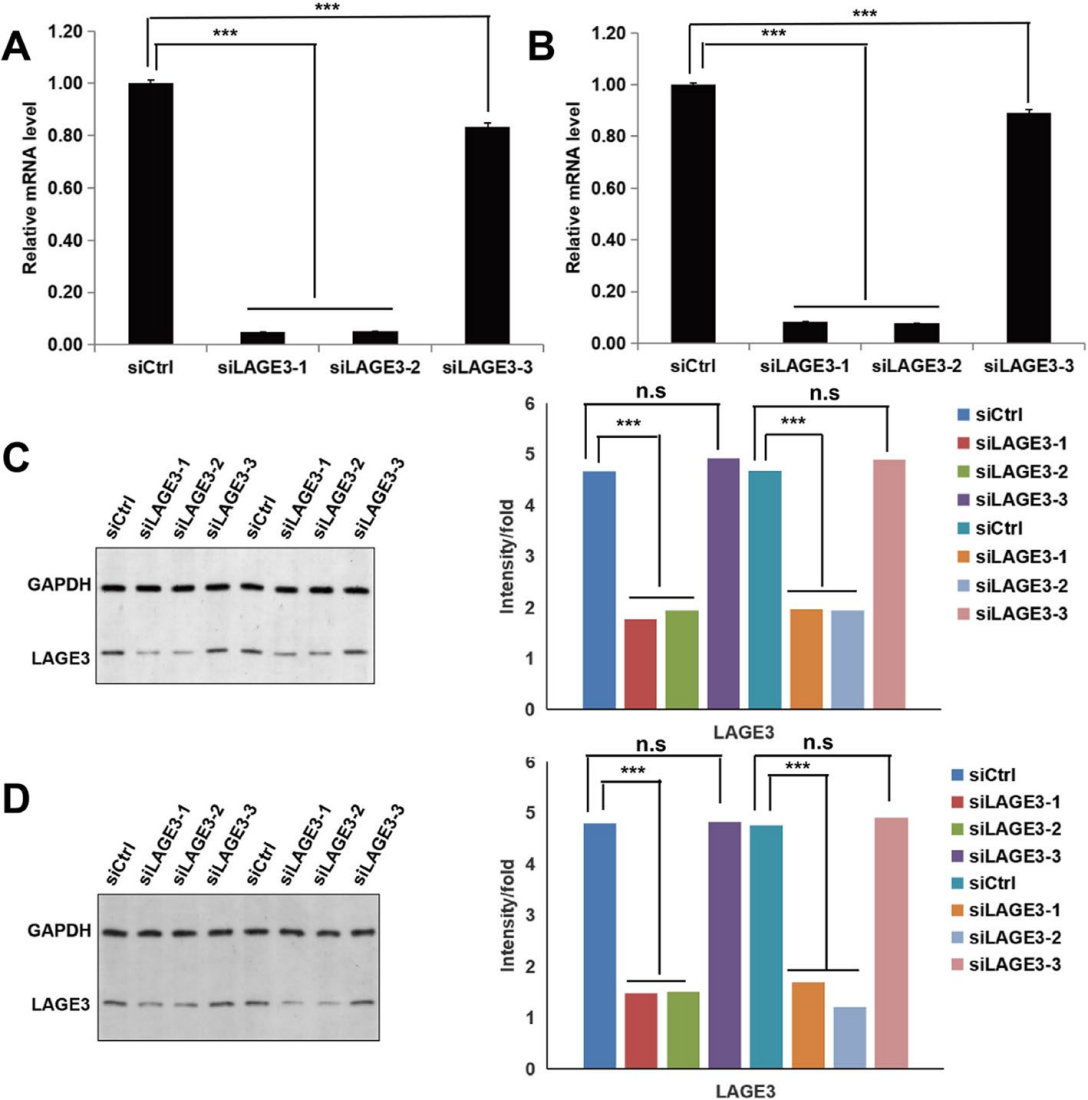
Silencing LAGE3 by infection of lentivirus-mediated siRNA to explore the effect of LAGE3 suppression on HCC progression. **A** and **C** The knockdown efficiency of LAGE3 by infection of lentivirus-mediated siRNA and the negative control siRNA in SMCC-7721 cells was verified by qRT-PCR and Western blot. Data were expressed as the mean ± s.d., *** *P* < 0.001. **B** and **D** The knockdown efficiency of LAGE3 by infection of lentivirus-mediated siRNA and the negative control siRNA in Huh-7 cells was verified by qRT-PCR and Western blot. Data were expressed as the mean ± s.d., *** *P* < 0.001, n.s: no significant difference

Furthermore, we explored the role of LAGE3 in the HCC progression both in vivo and in vitro, by initiating LAGE3 knockdown by transfecting LAGE3-specific siRNA plasmids. Then, the transfection efficiency of the three LAGE3-specific siRNA plasmids was examined while the qRT-PCR analysis results revealed that the mRNA expression levels of LAGE3 were significantly reduced in SMCC-7721 (Fig. 2A, *p* < 0.001) and Huh-7 (Fig. 2B, *p* < 0.001) cells transfected with LAGE3 specific siRNA-1 (siLAGE3–1), siRNA-2 (siLAGE3–2) and siRNA-3 (siLAGE3–3). In contrast, no significant difference was obtained in SMCC-7721 (Fig. 2C) and Huh-7 (Fig. 2D) cells transfected with LAGE3 specific siRNA-3 (siLAGE3–3) by Western blot analysis. Therefore, for subsequent molecular target therapy evaluations, SMCC-7721 and Huh-7 cell lines transfected with siLAGE3–1 or siLAGE3–2 were picked.

### LAGE3 knockdown inhibited the proliferation of HCC cells

**Fig. 3:**
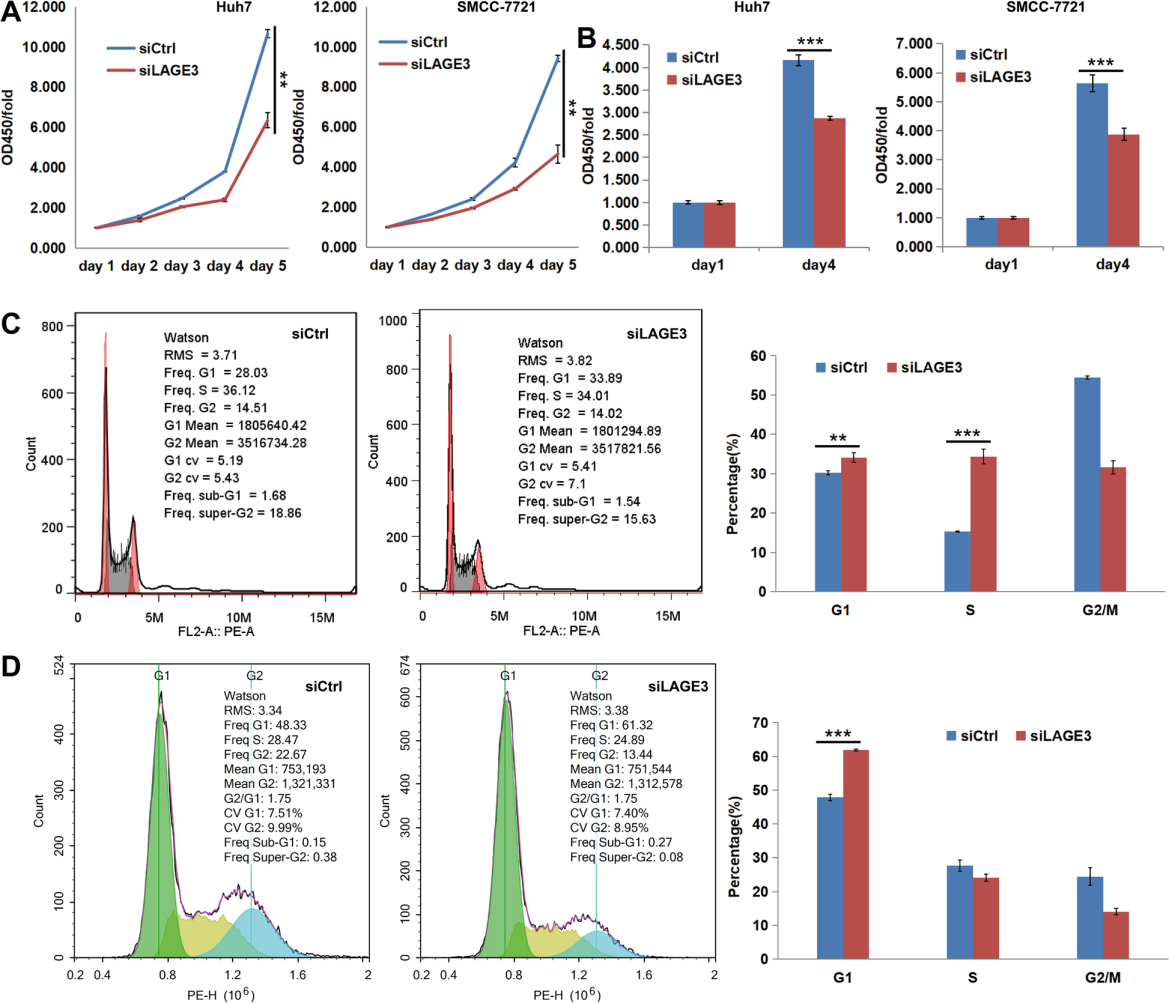
LAGE3 knockdown suppressed HCC cell proliferation. **A** The proliferation of SMCC-7721 and Huh-7 cells was significantly inhibited by LAGE3 knockdown in the CCK8 assay, and the data were expressed as the mean ± s. d., ** *p* < 0.01. **B** The proliferation of SMCC-7721 and Huh-7 cells was significantly inhibited by LAGE3 knockdown in BrdU assay, and the data were expressed as the mean ± s. d., *** *p* < 0.001. **C** The cycle of SMCC-7721 cells was significantly arrested in the S phase by LAGE3 knockdown while the data were expressed as the mean ± s. d., ** *p* < 0.01, *** *p* < 0.001. **D** The cycle of Huh-7 cells was significantly arrested in the G1 phase by LAGE3 knockdown while the data were expressed as the mean ± s. d., *** *p* < 0.001

Since LAGE3 positively promotes the proliferation of HCC cells and breast cancer cells [9, 11], our study involved CCK8 assay, BrdU assay, and cell cycle assay to explore the LAGE3 knockdown effects on the proliferation of HCC cells, and it was observed that the cell proliferation rate of the LAGE3-siRNA group was extraordinarily reduced in SMCC-7721 and Huh-7 cell lines when compared with the control group (Fig. 3A, B). Additionally, the cycle of the SMCC-7721 cell line (Fig. 3C) with LAGE3 knockdown was arrested in the G1 (*p* < 0.01) and S phase (*p* < 0.001), while the cycle of the Huh-7 cell line (Fig. 3D) with LAGE3 knockdown was arrested in the G1 phase (*p* < 0.001) when compared with the control cells. Therefore, it was evident that the cell cycle was arrested in a certain phase alone or G1 and S phase, in either case; thus, indicating that the knockdown of LAGE3 inhibits cellular proliferation.

### LAGE3 knockdown inhibited the migration and invasion of HCC cells

**Fig. 4:**
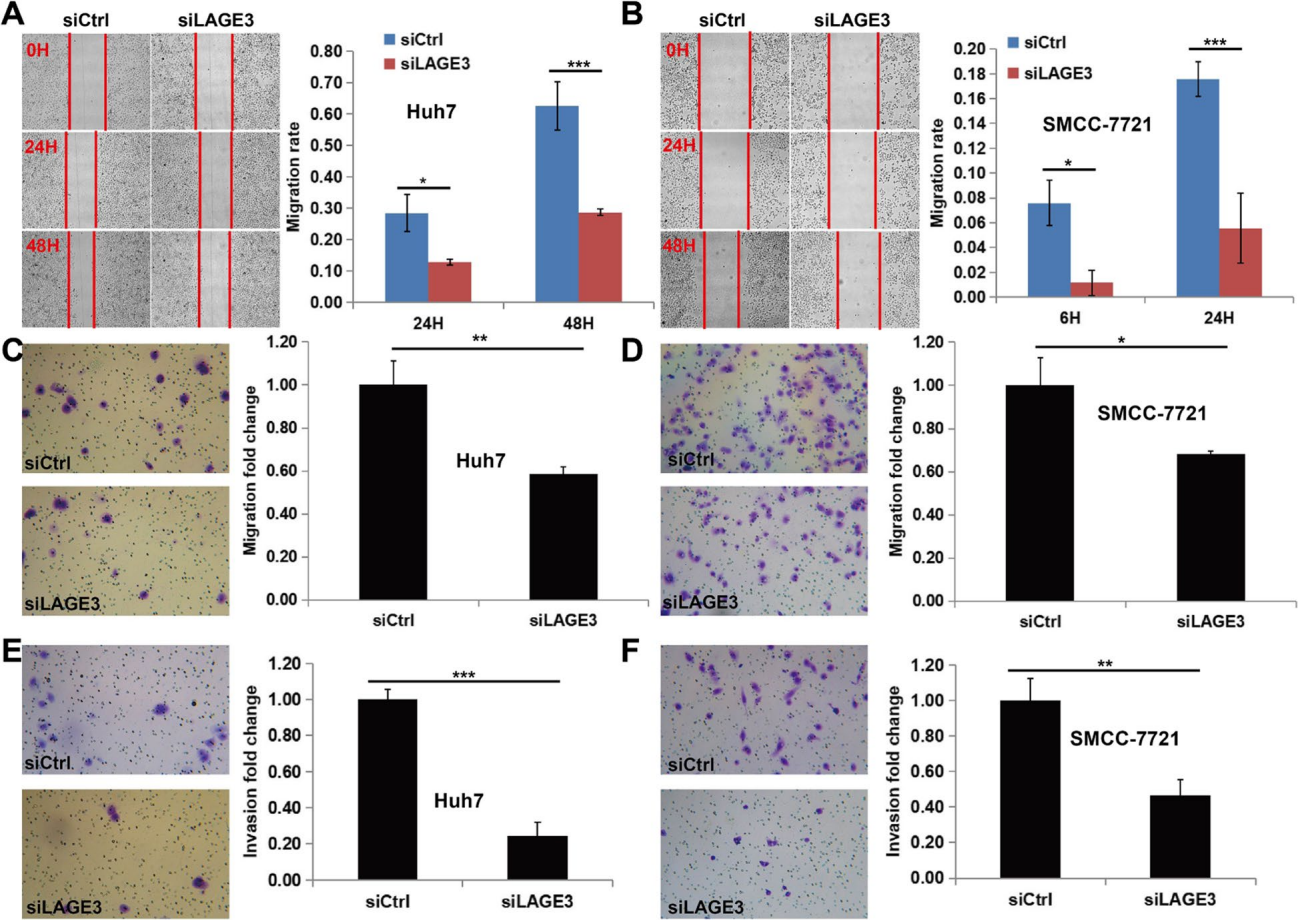
LAGE3 knockdown suppressed HCC cell migration and invasion. **A** and **B** Migration of Huh-7 and SMCC-7721 cells was significantly inhibited by LAGE3 knockdown in scratch healing assay expressing the data as the mean ± s. d., * *p* < 0.05 and *** *p* < 0.001. **C** and **D** Migration of Huh-7 and SMCC-7721 cells were significantly inhibited by LAGE3 knockdown in Transwell assay expressing the data as the mean ± s. d., * *p* < 0.05 and ** *p* < 0.01. **E** and **F** while the invasion of Huh-7 and SMCC-7721 cells were significantly inhibited by LAGE3 knockdown in Transwell assay expressing the data as the mean ± s. d., ** *p* < 0.01 and *** *p* < 0.001

Since LAGE3 positively promotes the migration and invasion of HCC cells and breast cancer cells [9, 11], migration and invasion of cancer cells are the key driving force for cancer metastasis and the main cause of cancer-related deaths [15, 16]. Therefore, scratch healing assay and Transwell assay were adopted to explore the effect of LAGE3 knockdown on the migration and invasion of HCC cells. It was observed that the cell migration rate of the LAGE3-siRNA group was extraordinarily reduced in SMCC-7721 and Huh-7 cell lines (Fig. 4A, B, C, and D**)** when compared with the control group while the invasion abilities of the SMCC-7721 and Huh-7 cell lines with LAGE3 knockdown were extraordinarily reduced as compared to the control cells (Fig. 4E and F). The above results also discovered that Huh-7 cells have a weaker migration ability, which can be inhibited more effectively by LAGE3 knockdown compared to SMCC-7721 cells.

### LAGE3 knockdown promoted apoptosis in HCC cells

**Fig. 5:**
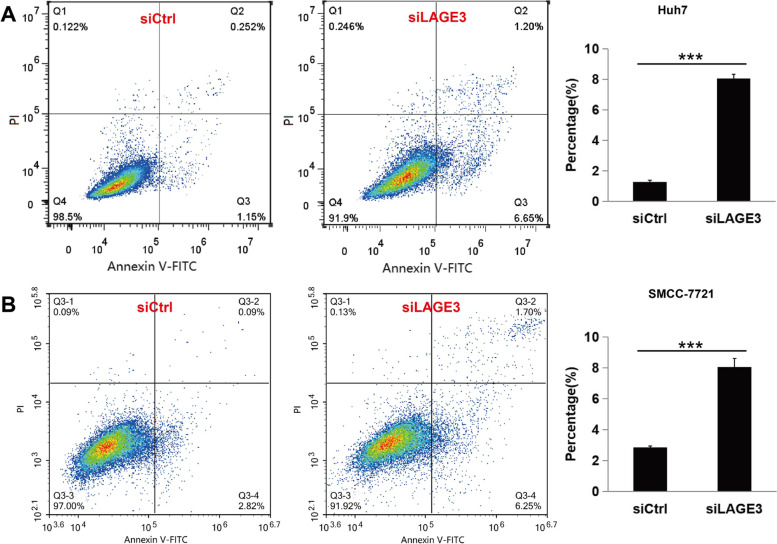
LAGE3 knockdown promoted HCC cells apoptosis. The proportion of apoptotic cells was significantly increased in the LAGE3 knockdown group compared with control cells in Huh7 (**A**) and SMCC-7721 (**B**) cell lines as assessed by flow cytometry thus expressing the data as the mean ± s. d., *** *p* < 0.001

The usage of Annexin V-FITC with PI inhibited PI from penetrating the cell membrane to stain the nuclei of living cells (Annexin V-FITC−/PI-) and early apoptotic cells (Annexin V-FITC+/PI-) but could stain late apoptotic cells and necrotic cells at the same time to show double-positive staining (Annexin V-FITC+/PI+). Furthermore, Annexin V-FITC/PI staining was applied to explore the role of LAGE3 knockdown on HCC cell apoptosis. It was discovered that the early apoptosis rate (6.65%), late apoptosis rate (1.20%), and necrosis rate (0.246%) of the LAGE3-siRNA group in the SMCC-7721 cell line were significantly enhanced as compared with the control group (Fig. 5A) while similar results were acquired in the Huh-7 cell line (Fig. 5B). The above results indicated that LAGE3 knockdown facilitates HCC cell apoptosis; thus, playing an important role in HCC progression.

### LAGE3 knockdown suppressed tumor growth in vivo

**Fig. 6:**
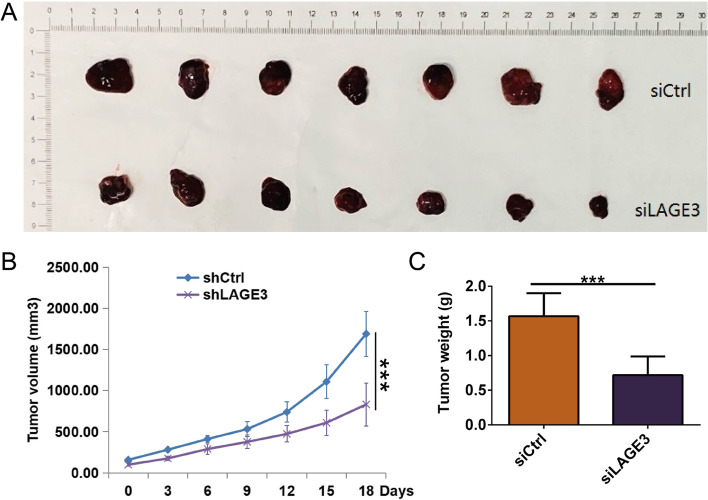
LAGE3 knockdown suppressed tumor growth in vivo. **A** and **B** The tumor volume was significantly decreased in the LAGE3 knockdown group while expressing the data as the mean ± s. d., *** *p* < 0.001. **C** The tumor weight was significantly reduced in the LAGE3 knockdown group while expressing the data as the mean ± s. d., *** *p* < 0.001

Since Huh7 cells transfected with siLAGE3 or siCtrl were subcutaneously inoculated into BALB/c nude mice in vivo, LAGE3 knockdown lessened the volume and weight of tumor tissues, respectively (Fig. 6A, B and C).

### The molecular mechanism of LAGE3 promotes the progression of HCC

**Fig. 7:**
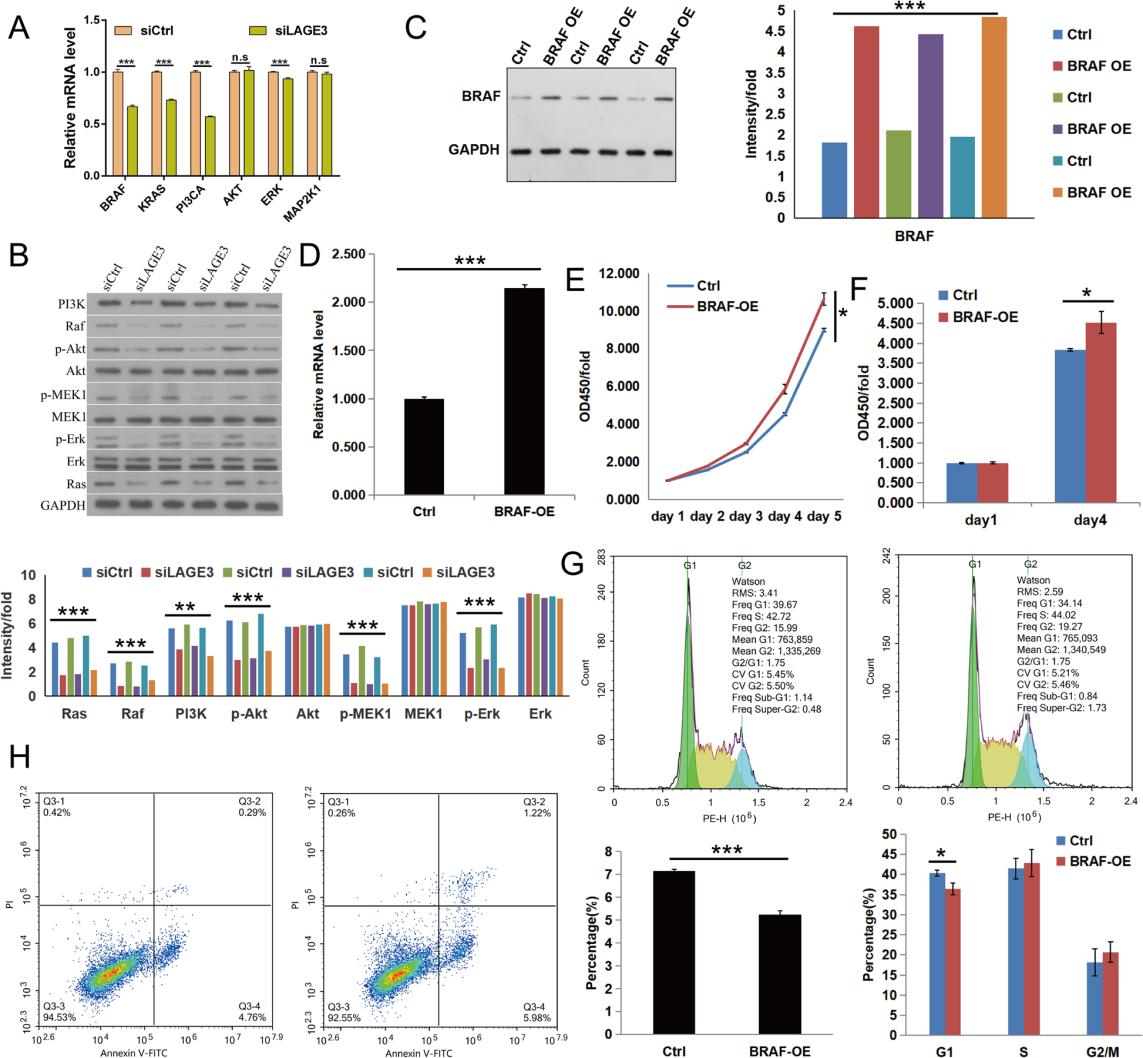
LAGE3 promotes the progression of HCC via PI3K/AKT/mTOR and Ras/RAF/MAPK pathways. **A** The mRNA levels of PI3CA, BRAF, AKT, MAP2K1, ERK, and KRAS in the siLAGE3 group were detected by qRT-PCR expressing the data as the mean ± s. d., n. s: no significant difference, *** *p* < 0.001. **B** The protein levels of PI3K, RAF, MUC1, p-Akt, p-MEK1, p-Erk, and Ras were significantly down-regulated in the siLAGE3 group while data were expressed as the mean ± s. d., ** *p* < 0.01 and *** *p* < 0.001. The overexpression efficiency of LAGE3 by LAGE3 gene lentivirus particles infection and the empty plasmid lentivirus particles in Huh7 cells was verified by Western blot (**C**) and qRT-PCR (**D**), where the data were expressed as the mean ± s. d., *** *p* < 0.001. The proliferation of Huh-7 cells was significantly enhanced in the overexpression of BRAF in (**E**) MTT assay and Brdu assay (**F**), while data were expressed as the mean ± s. d., * *p* < 0.05. **G** The Huh-7 cell cycle was significantly arrested in the S and G2/M phase in the BRAF overexpression, where the data were expressed as the mean ± s. d., * *p* < 0.05. **H** The proportion of apoptotic cells was significantly reduced in the BRAF overexpression group as assessed by flow cytometry and expressing the data as the mean ± s. d., * *p* < 0.05

Several studies have shown that abnormal activation of PI3K/AKT/mTOR and Ras/RAF/MAPK pathways are beneficial to tumor cell proliferation, migration, and invasion; thus, acting as an important entity in tumor development and drug resistance [17–19]. In this study, Western blot and qRT-PCR tests were used to verify the mRNA and protein expression levels of key molecular nodes in both PI3K/AKT/mTOR and Ras/RAF/MAPK pathways in Huh-7 cell lines, which are displayed in (Fig. 7A and B**)**, when compared with the siCtrl group. It was discovered that PI3CA, BRAF, ERK, and KRAS in the siLAGE3 group were significantly down-regulated at the mRNA level and the protein levels of PI3K, RAF p-Akt, p-MEK1, p-Erk, and Ras were also considerably down-regulated. The influence of these genes and proteins on HCC, after BRAF overexpression in Huh-7 cells (Fig. 7C and D), was determined by investigating the proliferation as well as the anti-apoptotic ability of Huh-7 cells and is displayed in (Fig. 7E, F, G, and H**)** and it was observed that the BRAF overexpression enhances the proliferation ability of HCC cells, while arresting the cells in the S and G2/M phase, and thus reducing the ability of cellular apoptosis.

## Discussion

Hepatocellular carcinoma (HCC) is one of the most aggressive and lethal malignancies as well as a leading cause of carcinoma-associated mortality worldwide and has left a huge negative impact on human health [20, 21]. Moreover, coupled with the timely recognition of its severity and the deleterious propensity, several new effective treatment regimens have emerged for the treatment of HCC, including liver transplantation for early HCC patients, transarterial chemoembolization (TACE) for intermediate-stage HCC patients, and systemic drug therapy for advanced HCC patients [22–24]. Although several improvements have occurred in various non-drug modalities and pharmacotherapies for HCC, the overall survival rate of patients is still far from satisfactory [25, 26]. In addition, despite the improvement in therapies by the immune checkpoint inhibitors, the mortality rate of HCC is still high [27–30]. Although there are numerous ongoing pre-clinical studies on HCC treatment, consistent progress in molecular targeted therapies and other signaling pathway targets seeking newer drugs might prove beneficial for HCC treatment [31–34].

Many previous studies suggested that an abnormal LAGE3 expression was observed in an array of cells and organs undergoing malignant transformation [5–7, 9, 35, 36]. In our study, TCGA database analysis revealed that when compared to normal liver tissues, an abnormally high level of LAGE3 expression was observed in LIHC tissues that indicated a poor survival prognosis for high LAGE3 expression patients; thus, pointing that LAGE3 may be a vital triggering factor in tumorigenesis and development of HCC. Furthermore, a qRT-PCR analysis verified an abnormally high LAGE3 expression in four HCC cell lines, especially in SMCC-7721 and Huh7 cell lines, which were then demarcated for subsequent gene-expression data evaluations to identify potential therapeutic targets. The results of tissue chip staining showed that LAGE3 was highly expressed in most liver cancer tissues, but the expression rate still did not meet our expectations. This may be related to the limited number of specimens, different clinical stages, and differences in the treatment of patients. Therefore, it is suggested that a clinically large sample follow-up is required to provide more accurate mean values and a smaller margin of error.

The malignant progression of a tumor encompasses various biological processes involving cellular proliferation, migration, invasion, and anti-apoptotic cascade. A bioinformatics analysis study by Dong X et al. revealed that since LAGE3 is highly expressed in breast cancer tissues, it can independently predict the survival of breast cancer patients. In contrast, the inhibition of LAGE3 expression can restrain the proliferation, migration, and invasion of triple-negative breast cancer cell lines and induce their apoptosis in vitro [9]. In our study, several lentiviral plasmids were applied to transfect HCC cells for silencing the LAGE3 gene, while qRT-PCR and western blot assays were procured to select the best knockdown efficiency and a good survival status cell line for the subsequent functional molecular target study.

Our in vitro CCK8 assay and BrdU assay study results disclosed that LAGE3 gene knockdown can reduce the proliferation of HCC as the cell cycle assay showed that the LAGE3 gene knockdown in SMCC-7721 cells arrested the cells in the S phase while the Huh7 cells in the G1 phase were arrested. Henceforth the cell cycle was arrested in a certain phase alone in either case, indicating that cellular proliferation had been suppressed. Therefore, the above results proved that LAGE3 knockdown inhibited cellular proliferation. Similarly, it was observed that LAGE3 knockdown could inhibit the migration, invasion, and cause apoptosis induction in HCC cells and effectively inhibit the growth of tumor tissues in vivo. The above results comprehensively stated that LAGE3 can regulate the metastasis and malignant progression of HCC.

As the PI3K/AKT/mTOR signaling pathway is interconnected to several cell growth and survival aspects, it is frequently activated in various cancers and can be precisely evaluated as an optimistic therapeutic target [17, 37, 38]. Upstream molecular switches, including receptor tyrosine kinases and Ras regulate several positive regulators like PI3K, AKT, mTOR, and eIF4E, which are abnormally activated in many tumorigenesis processes and are involved in controlling the survival, proliferation, and migration of cancer cells [37]. The oncogenic effect of the PI3K/AKT/mTOR axis might be achieved by stimulating cellular proliferation, survival, migration, and invasion while simultaneously inhibiting autophagy and aging processes [19, 37, 39]. The MAPK cascade can regulate cellular processes such as cell proliferation, migration, apoptosis, and stress response [18, 40], while overexpression of ERK in tumor cells can appropriately accelerate the tumorigenesis and progression of cancer while negatively affecting the cancer patient’s prognosis [18, 41]. It is a well-known fact that the Ras/RAF/MAPK (MEK)/ERK pathway is the most prominent signal axis in all MAPK signal pathways, which plays a vital role in the initiation, survival, and progression of malignant cells [18, 42]. Therefore, our study investigated the key node molecules’ mRNA and protein expression levels in both PI3K/AKT/mTOR and Ras/RAF/MAPK pathways in HCC cells after LAGE3 gene knockdown. Our study results observed that LAGE3 knockdown can effectively inhibit the abnormal expression of the node as mentioned above molecules, thereby suggesting that both PI3K/AKT/mTOR and Ras/RAF/MAPK pathways might be involved in the regulation of HCC metastasis and malignant progression. Our study results further verified the effect of abnormal activation of the two pathways mentioned above in HCC cells and observed the BRAF overexpression enhancing the proliferation and apoptosis of HCC cells; thus, stating that overexpression of BRAF can promote the proliferation and anti-apoptotic ability of HCC cells.

In conclusion, it could be stated that LAGE3 has prognostic prediction value and may influence HCC tumor progression by promoting the proliferation, survival, migration, invasion, and anti-apoptotic ability of HCC cells through PI3K/AKT/mTOR and Ras/RAF/MAPK pathways. Therefore, LAGE3 could be a feasible prognostic biomarker and an effective target for HCC treatment interventions.

## Conclusion

Our study results displayed that since LAGE3 might promote tumor development in HCC via PI3K/AKT/mTOR and Ras/RAF/MAPK pathways, the development of LAGE3 target drugs may be a new treatment strategy for HCC patients. Furthermore, all necessary efforts should be undertaken to merge correlative studies in ongoing pre-clinical and clinical trials so that several promising therapeutic targets in related taxonomic signaling pathways might guide new trends in the HCC treatment.

## Supplementary Information

**Additional file 1.**

## Data Availability

The data that support the findings of this study are available from the corresponding author upon reasonable request.

## Abbreviations

HCC: Hepatocellular carcinoma
LAGE3: L antigen family member 3
EMT: Epithelial-mesenchymal transition
PTC: Papillary thyroid carcinoma
CM: Melanoma
BC: Breast cancer
LIHC: Liver hepatocellular carcinoma
