# Supplementary material for: Correlation of LAGE3 with unfavorable prognosis and promoting tumor development in HCC via PI3K/AKT/mTOR and Ras/RAF/MAPK pathways
Source: BMC Cancer. 2022 Mar 21;22:298. doi: 10.1186/s12885-022-09398-3 (PMC8939149; doi:10.1186/s12885-022-09398-3)
Supplement: Supplementary file 1 — Additional file 1. [file 12885_2022_9398_MOESM1_ESM.zip › RPN810_INSTRUCTION_02.PDF]

product code

RPN810

ECL DualVue Western Blotting Markers

Molecular weight range 15–150 kDa

Before using this product, please read the instructions for safe handling

Form

ECL DualVue Western blotting markers are supplied in 35% glycerol and sample buffer containing mercaptoethanesulphonic acid (MESNA) as a reducing agent(2).

Warning

For research use only.

Not recommended or intended for diagnosis of disease in humans or animals.

Do not use internally or externally in humans or animals.

Handling

Storage

Store S-protein-HRP conjugate at -15 °C to -30 °C.

Store DualVue Western blotting markers at -15 °C to -30 °C.

Ensure conjugate and markers are returned to the freezer immediately after each use.

Expiry

Stable for three months when stored under recommended conditions.

Components

- 125 µl ECL DualVue™ Western blotting markers
- 50 µl S-protein-HRP conjugate

The kit contains sufficient components for marker detection on 25 blots (10 cm × 10 cm; 10 ml incubation volumes using Hybond™ECL™ nitrocellulose membrane in conjunction with ECL detection reagents).

Safety warnings and precautions

Warning: For research use only. Not recommended or intended for diagnosis of disease in humans or animals. Do not use internally in humans or animals.

All chemicals should be considered as potentially hazardous. We therefore recommend that this product is handled only by those persons who have been trained in laboratory techniques and that it is used in accordance with the principles of good laboratory practice. Wear suitable protective clothing such as laboratory overalls, safety glasses and gloves. Care should be taken to avoid contact with skin or eyes. In the case of contact with skin or eyes wash immediately with water. See material safety data sheet(s) and/or safety statement(s) for specific advice.

This product is used in conjunction with gel electrophoresis. Please follow the manufacturers instructions relating to the handling and use of the equipment and materials.

Description

ECL DualVue Western blotting markers consist of a combination of two types of protein marker:

- Pre-stained indicator proteins that confirm blot transfer and blot orientation
- Recombinant tagged proteins that ensure accurate molecular mass determination of the target protein(s) following chemiluminescent detection on film or by CCD imaging

There are three pre-stained indicator proteins with approximate molecular masses of 15 kDa (red), 16 kDa (blue) and 100 kDa (red). These three coloured bands are clearly visible following transfer to membrane providing a reliable indicator of protein transfer (see Figure 1A). Also, blot orientation is apparent at all times. These proteins are not tagged and therefore not subsequently detected by chemiluminescent substrates.

These indicator proteins are supplemented with a set of seven recombinant protein markers with precise molecular masses that each contain a tagged peptide sequence. The marker set is easily and specifically detected by binding S-protein-HRP conjugate and developing with chemiluminescent substrates. Since no chemical modification is required to label the marker proteins, their migration accurately represents their sizes when separated on a polyacrylamide gel as described by Laemmli(1).

The molecular masses of the recombinant tagged proteins are 15, 25, 35, 50, 75, 100 and 150 kDa (see Figure 1B). The migration and band sharpness of the recombinant tagged proteins are unaffected by the presence of the pre-stained indicator proteins.

ECL DualVue Western blotting markers cannot be used in conjunction with full range Rainbow™ markers (catalogue code RPN800) since the proteins used for this product also contain the tagged region. However, the product is compatible with high or low range Rainbow markers (catalogue codes RPN756 and RPN755 respectively).

Protocol

The S-protein-HRP conjugate can be added during either the primary or secondary antibody incubation. The protocol below includes addition of the conjugate during the secondary antibody incubation.

1. Remove the ECL DualVue Western blotting markers from storage at -15 °C to -30 °C and allow equilibration to room temperature. A precipitate of SDS may form on storage at -15 °C to -30 °C. If necessary briefly warm the solution at 37 °C to dissolve the precipitate.

2. Mix well and add 5 µl of marker to an equal volume of loading buffer containing 10% Beta-mercaptoethanol (or a loading buffer containing an equivalent reducing agent). Perform electrophoresis according to standard techniques.

3. Transfer the proteins electrophoretically to Hybond ECL or Hybond PVDF for optimum results. Any standard blotting device can be used according to the manufacturer's instructions. The transferred pre-stained marker proteins should be visible on the membrane after transfer.

4. Process the blot according to your standard protocol for blocking and primary antibody incubation steps.

5. Incubate the membrane with your secondary antibody at the required dilution. To this solution add the S-protein-HRP conjugate at the appropriate dilution for the system being used (as indicated in Table 1). A minimum incubation of 30 minutes at room temperature is recommended for maximum signal generation.

6. Wash the membrane according to standard protocols.

7. Visualize the proteins using chemiluminescent substrates according to manufacturer's instructions. Initial film exposure times of 1 and 2.5 minutes are recommended.

Table 1. Recommended S-protein-HRP conjugate dilutions when using Amersham Biosciences chemiluminescent detection reagents with Hybond membranes and detection on film.

Chemiluminescent Detection Reagent	Hybond ECL Nitrocellulose Membrane	Hybond-P PVDF Membrane
ECL™	1/5 000	1/10 000
ECL Plus™	1/10 000	1/20 000
ECL *Advance*™	1/100 000	1/200 000

NOTE: For CCD camera detection the S-protein-HRP conjugate concentration needs to be determined empirically - as a guide a 10-fold increase in concentration is recommended.

Typical Results

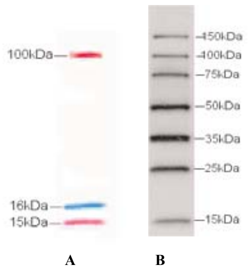

Figure 1.

ECL DualVue Western Blotting markers after electrophoresis on a 4-20% SDS-PAGE slab gel and transfer onto Hybond ECL nitrocellulose membrane showing pre-stained indicator proteins (**A**) and tagged proteins after detection with ECL Western blotting detection reagents (**B**). Electrophoresis performed for 45 minutes at 200 volts, 1 minute film exposure.

Quality Control

Each batch of ECL DualVue Western blotting markers is assessed for colour intensity of the pre-stained protein markers on transfer to membrane. The tagged proteins are assessed for band integrity and intensity by detection with ECL on Hybond ECL nitrocellulose membrane.

The 15 and 150 kDa bands may give weaker signals than the other species depending on the percentage gel, membrane support or transfer conditions used.

Under certain conditions a very weak pre-stained protein band at 35 kDa may be observed on the membrane following transfer.

Related products

Amersham Biosciences offers a comprehensive range of Western blotting reagents and hardware all with proven compatibility to ensure reproducible high quality results. For a complete listing of products available see the current Amersham Biosciences catalogue or visit our web site at www.amershambiosciences.com.

RPN2106	ECL Western Blotting Detection Reagents for 4000 cm^2 membrane
RPN2132	ECL Plus Western Blotting Detection Reagents for 1000 cm^2 membrane
RPN2135	ECL *Advance* Western Blotting Detection Kit<br>Other pack sizes and detection reagents also available
RPN755	Low-range Rainbow MW markers
RPN756	High-range Rainbow MW markers
RPN800	Full-range Rainbow MW markers (recombinant)
RPN2107	ECL Western Blotting MW markers, biotinylated
RPN2020F	Hybond-P membrane (PVDF, pore size 0.45 µm)
RPN303D	Hybond-ECL membrane (nitrocellulose, pore size 0.45 µm)<br>Other membrane sizes also available
RPN2103K	Hyperfilm™ ECL 18 × 24 cm, pack of 25 films<br>Other film sizes are also available
RPN1051	Streptavidin-biotinylated horseradish peroxidase complex
RPN1231	Streptavidin horseradish peroxidase conjugate
NA931	Mouse IgG, HRP linked whole antibody (from sheep), 1 ml
NA934	Rabbit IgG, HRP linked whole antibody (from sheep), 1 ml<br>Other conjugates are also available

References

1. Laemmli, U.K. *Nature,* **227**, 681 (1970).
2. Singh, R. *Biotechniques,* **17**, 263 (1994).

Legal

ECL *Advance*, ECL DualVue, ECL, ECL Plus, Rainbow, Hyperfilm and Hybond are trademarks of Amersham Biosciences Limited

Amersham and Amersham Biosciences are trademarks of Amersham plc

All goods and services are sold subject to the terms and conditions of sale of the company within the Amersham Biosciences Group which supplies them. A copy of these terms and conditions is available on request.

©Amersham Biosciences UK Limited 2003 - All rights reserved

Product information

Product name	code
ECL DualVue Western Blotting Markers	RPN810

Related products

See listed related products

http://www.amershambiosciences.com

Amersham Biosciences UK Limited
Amersham Place Little Chalfont Buckinghamshire UK HP7 9NA

Amersham Biosciences AB
SE-751 84 Uppsala Sweden

Amersham Biosciences Corp
800 Centennial Avenue PO Box 1327 Piscataway NJ 08855 USA

Amersham Biosciences Europe GmbH
Munzinger Strasse 9 D-79111 Freiburg Germany

Amersham Biosciences
