# Supplementary material for: Correlation of LAGE3 with unfavorable prognosis and promoting tumor development in HCC via PI3K/AKT/mTOR and Ras/RAF/MAPK pathways
Source: BMC Cancer. 2022 Mar 21;22:298. doi: 10.1186/s12885-022-09398-3 (PMC8939149; doi:10.1186/s12885-022-09398-3)
Supplement: Supplementary file 1 — Additional file 1. [file 12885_2022_9398_MOESM1_ESM.zip › Supplementary Datas.pdf]

The socio-demographic and clinical-pathological features of the HCC patients and controls

Location	Age	Gender	Pathological diagnosis	Grade	TNM	Stage	Organization type	Primary/ transfer	Lymph node metastasis
A1	44	Male	HCC	1	T2N0M0	II	Malignant	Primary	No
A2	69	Female	HCC	1	T2N0M0	II	Malignant	Primary	No
A3	53	Male	HCC	1	T2N0M0	II	Malignant	Primary	No
A4	44	Male	HCC	2	T2N0M0	II	Malignant	Primary	No
A5	40	Male	HCC	1	T2N0M0	II	Malignant	Primary	No
A6	72	Male	HCC	2	T2N0M0	II	Malignant	Primary	No
A7	59	Male	HCC	1--2	T2N0M0	II	Malignant	Primary	No, 0/10
A8	48	Male	HCC	1--2	T2N0M0	II	Malignant	Primary	No
A9	52	Male	HCC	2	T2N0M0	II	Malignant	Primary	No
A10	33	Male	HCC	2	T3N0M0	IIIA	Malignant	Primary	No
A11	45	Female	HCC	2	T2N0M0	II	Malignant	Primary	No
A12	19	Male	HCC	2	T2N0M0	II	Malignant	Primary	No
B1	65	Female	HCC	2	T2N0M0	II	Malignant	Primary	No

B2	48	Male	HCC	1	T2N0M0	II	Malignant	Primary	No
B3	45	Male	HCC	2	T2N0M0	II	Malignant	Primary	No
B4	49	Male	HCC	2	T2N0M0	II	Malignant	Primary	No
B5	49	Male	HCC	2	T2N0M0	II	Malignant	Primary	No
B6	25	Male	HCC	3	T3N0M0	IIIA	Malignant	Primary	No
B7	59	Female	HCC	2	T3N0M0	IIIA	Malignant	Primary	No
B8	42	Male	HCC	2	T2N0M0	II	Malignant	Primary	No
B9	68	Female	HCC	3	T3N0M0	III	Malignant	Primary	No
B10	76	Male	HCC	2	T3N0M0	IIIA	Malignant	Primary	No
B11	30	Male	HCC	2	T2N0M0	II	Malignant	Primary	No
B12	58	Male	HCC	3	T2N0M0	II	Malignant	Primary	No
C1	68	Male	HCC	2	T3N0M0	IIIA	Malignant	Primary	No
C2	65	Male	HCC	2	T2N0M0	II	Malignant	Primary	No
C3	44	Male	HCC	1	T2N0M0	II	Malignant	Primary	No
C4	40	Male	HCC	1	T2N0M0	II	Malignant	Primary	No
C5	38	Male	HCC	2	T2N0M0	II	Malignant	Primary	No
C6	56	Male	HCC	2	T2N0M0	II	Malignant	Primary	No
C7	57	Male	HCC	2	T2N0M0	II	Malignan	Primary	No

							t		
C8	34	Fem ale	HCC	2	T3N0M0	III A	Malignan t	Primary	No
C9	50	Male	HCC	2	-		Malignan t	Primary	No
C1 0	50	Male	HCC	2	T2N0M0	II	Malignan t	Primary	No
C1 1	32	Male	HCC	2	T3N0M0	III A	Malignan t	Primary	No
C1 2	45	Male	HCC	2--3	T3N0M0	III A	Malignan t	Primary	No
D1	59	Male	HCC	1	-		Malignan t	Primary	No
D2	60	Male	HCC	1--2	-		Malignan t	Primary	No
D3	50	Fem ale	HCC	2	T2N0M0	II	Malignan t	Primary	No
D4	45	Male	HCC	2	T3N0M0	III A	Malignan t	Primary	No
D5	55	Male	HCC	2	T4N0M0	III C	Malignan t	Primary	No
D6	41	Fem ale	HCC	3	T3N0M0	III A	Malignan t	Primary	No
D7	45	Male	HCC	2	T2N0M0	II	Malignan t	Primary	No
D8	51	Fem ale	HCC	2	T3N0M0	III A	Malignan t	Primary	No
D9	43	Male	HCC	2	T4N0M0	III C	Malignan t	Primary	No

D10	47	Male	HCC	3	T3N0M0	IIIA	Malignant	Primary	No
D11	56	Male	HCC	2	T2N0M0	II	Malignant	Primary	No
D12	40	Male	HCC	2	T2N0M0	II	Malignant	Primary	No
E1	76	Male	HCC	2	T3N1M0	IVA	Malignant	Primary	Yes
E2	36	Male	HCC	1	T3N0M0	IIIA	Malignant	Primary	No
E3	56	Female	HCC	2	T3N0M0	IIIA	Malignant	Primary	No
E4	45	Male	HCC	2	T3N0M0	IIIA	Malignant	Primary	No
E5	63	Male	HCC	2	T3N0M0	IIIA	Malignant	Primary	No, 0/2
E6	45	Male	HCC	2--3	T2N0M0	II	Malignant	Primary	No
E7	62	Male	HCC	2	T2N0M0	II	Malignant	Primary	No
E8	61	Male	HCC	2	T2N0M0	II	Malignant	Primary	No
E9	45	Male	HCC	3	T3N0M0	IIIA	Malignant	Primary	No
E10	64	Male	HCC	2	T3N1M0	IVA	Malignant	Primary	Hilar lymph node metastasis
E11	51	Male	HCC	2--3	T2N0M0	II	Malignant	Primary	No

E12	59	Male	HCC	2	T2N0M0	II	Malignant	Primary	No
F1	48	Male	HCC	2	T3N0M0	IIIA	Malignant	Primary	No
F2	37	Male	HCC	3	T3N0M0	IIIA	Malignant	Primary	No
F3	37	Male	HCC	2--3	T2N0M0	II	Malignant	Primary	No
F4	64	Male	HCC	2	T3N0M0	IIIA	Malignant	Primary	No
F5	42	Male	HCC	3	T2N0M0	II	Malignant	Primary	No
F6	54	Male	HCC	3	-	-	Malignant	Primary	No
F7	64	Male	HCC	2	T3N0M0	IIIA	Malignant	Primary	No
F8	71	Male	HCC	3	T2N0M0	II	Malignant	Primary	No
F9	52	Female	HCC	2	T2N0M0	II	Malignant	Primary	No
F10	42	Male	HCC	2	T2N0M0	II	Malignant	Primary	No
F11	65	Male	HCC	2	T2N0M0	II	Malignant	Primary	No
F12	42	Male	HCC	2	T4N0M0	IIIC	Malignant	Primary	No
G1	48	Male	HCC	3	T3N0M0	III	Malignant	Primary	No
G2	45	Male	HCC	3	T2N0M0	II	Malignant	Primary	No

G3	48	Male	HCC	3	T3N0M0	III A	Malignant	Primary	No
G4	52	Male	HCC	2--3	T3N0M0	III A	Malignant	Primary	No
G5	55	Male	HCC	3	T3N0M0	III A	Malignant	Primary	No
G6	56	Male	HCC	3	T3N0M0	III A	Malignant	Primary	No
G7	21	Male	HCC	3	T3N0M0	III A	Malignant	Primary	No
G8	48	Male	Liver cirrhosis		-	-	Paracancerous	Primary	No
G9	62	Male	Cirrhosis of the liver with cholestasis		-	-	Paracancerous	Primary	No
G10	50	Male	Liver cirrhosis		-	-	Paracancerous	Primary	No
G11	52	Male	Liver cirrhosis		-	-	Paracancerous	Primary	No
G12	45	Male	Liver cirrhosis		-	-	Benign	Primary	-
H1	56	Male	Liver cirrhosis		-	-	Benign	Primary	-

H2	65	Male	Liver cirrhosis		-	-	Benign	Primary	-
H3	48	Male	Liver cirrhosis		-	-	Benign	Primary	-
H4	52	Male	Liver cirrhosis		-	-	Benign	Primary	-
H5	55	Male	Liver cirrhosis		-	-	Benign	Primary	-
H6	56	Fem ale	Liver tissue		-	-	Normal	-	-
H7	23	Male	Liver tissue		-	-	Normal	-	-
H8	58	Male	Liver tissue		-	-	Normal	-	-
H9	35	Male	Liver tissue		-	-	Normal	-	-
H1 0	25	Fem ale	Liver tissue		-	-	Normal	-	-
H1 1	50	Male	Liver tissue		-	-	Normal	-	-

HCC: Hepatocellular carcinoma
