# Supplementary material for: Correlation of LAGE3 with unfavorable prognosis and promoting tumor development in HCC via PI3K/AKT/mTOR and Ras/RAF/MAPK pathways
Source: BMC Cancer. 2022 Mar 21;22:298. doi: 10.1186/s12885-022-09398-3 (PMC8939149; doi:10.1186/s12885-022-09398-3)
Supplement: Supplementary file 1 — Additional file 1. [file 12885_2022_9398_MOESM1_ESM.zip › Supplementary materials.pdf]

LAGE3 Correlates With Unfavorable Prognosis And Promotes Tumor Development In HCC Via PI3K/AKT/mTOR And RAS/RAF/MAPK Pathway

Yun Li, Hui Xiong*

Department of General Surgery, the First Affiliated Hospital of Nanchang University, Nanchang 330000, China

Corresponding author: Hui Xiong, Department of General Surgery, the First Affiliated Hospital of Nanchang University, Nanchang 330000, China. E-mail: huixiong3601@163.com

BRAF overexpression sequence design

Item Number: NM_004333.5

Gene name: BRAF

Sequence length: 2301

Vector name: pCDH-CMV-MCS-EF1-copGFP-T2A-Puro

Fluorescent label: copGFP

Resistance label: Puro

5'cloning site: Xba I

3'cloning site: NotI

Destination sequence:

5'-
ATGGCGGCGCTGAGCGGTGGCGGTGGTGGCGGCGCGGAGCCGGGCCAGGCTCTGTTCAACGGGGACATG
GAGCCCGAGGCCGGCGCCGGCGCCGGCGCCGCGGCCTCTTCGGCTGCGGACCCTGCCATTCCGGAGGAG
GTGTGGAATATCAAACAAATGATTAAGTTGACACAGGAACATATAGAGGCCCTATTGGACAAATTTGGTGGG
GAGCATAATCCACCATCAATATATCTGGAGGCCTATGAAGAATACACCAGCAAGCTAGATGCACTCCAACAAA
GAGAACAACAGTTATTGGAATCTCTGGGGAACGGAACTGATTTTTCTGTTTCTAGCTCTGCATCAATGGATAC
CGTTACATCTTCTTCCTCTTCTAGCCTTTCAGTGCTACCTTCATCTCTTTCAGTTTTTCAAAATCCCACAGATGT
GGCACGGAGCAACCCCAAGTCACCACAAAAACCTATCGTTAGAGTCTTCCTGCCCAACAAACAGAGGACAG
TGGTACCTGCAAGGTGTGGAGTTACAGTCCGAGACAGTCTAAAGAAAGCACTGATGATGAGAGGTCTAATC
CCAGAGTGCTGTGCTGTTTACAGAATTCAGGATGGAGAGAAGAAACCAATTGGTTGGGACACTGATATTTC
CTGGCTTACTGGAGAAGAATTGCATGTGGAAGTGTTGGAGAATGTTCCACTTACAA
CACACAACTTTGTACGAAAAACGTTTTTCACCTTAGCATTTTGTGACTTTTGTCGAAAGCTGCTTTTCCAGGG
TTTCCGCTGTCAAACATGTGGTTATAAATTTCACCAGCGTTGTAGTACAGAAGTTCCACTGATGTGTGTTAATT
ATGACCAACTTGATTTGCTGTTTGTCTCCAAGTTCTTTGAACACCACCCAATACCACAGGAAGAGGCGTCCTT

AGCAGAGACTGCCCTAACATCTGGATCATCCCCTTCCGCACCCGCCTCGGACTCTATTGGGCCCCAAATTCTC
ACCAGTCCGTCTCCTTCAAAATCCATTCCAATTCCACAGCCCTTCCGACCAGCAGATGAAGATCATCGAAATC
AATTTGGGCAACGAGACCGATCCTCATCAGCTCCCAATGTGCATATAAACACAATAGAACCTGTCAATATTGA
TGACTTGATTAGAGACCAAGGATTTCGTGGTGATGGAGGATCAACCACAGGTTTGTCTGCTACCCCCCCTGC
CTCATTACCTGGCTCACTAACTAACGTGAAAGCCTTACAGAAATCTCCAGGACCTCAGCGAGAAAGGAAGTC
ATCTTCATCCTCAGAAGACAGGAATCGAATGAAAACACTTGGTAGACGGGACTCGAGTGATGATTGGGAGA
TTCCTGATGGGCAGATTACAGTGGGACAAAGAATTGGATCTGGATCATTTGGAACAGTCTACAAGGGAAAG
TGGCATGGTGATGTGGCAGTGAAAATGTTGAATGTGACAGCACCTACACCTCAGCAGTTACAAGCCTTCAAA
AATGAAGTAGGAGTACTCAGGAAAACACGACATGTGAATATCCTACTCTTCATGGGCTATTCCACAAAGCCA
CAACTGGCTATTGTTACCCAGTGGTGTGAGGGCTCCAGCTTGTATCACCATCTCCATATCATTGAGACCAAAT
TTGAGATGATCAAACTTATAGATATTGCACGACAGACTGCACAGGGCATGGATTACTTACACGCCAAGTCAAT
CATCCACAGAGACCTCAAGAGTAATAATATATTTCTTCATGAAGACCTCACAGTAAAAATAGGTGATTTTGGT
CTAGCTACAGTGAAATCTCGATGGAGTGGGTCCCATCAGTTTGAACAGTTGTCTGGATCCATTTTGTGGATG
GCACCAGAAGTCATCAGAATGCAAGATAAAAATCCATACAGCTTTCAGTCAGATGTATATGCATTTGGAATTG
TTCTGTATGAATTGATGACTGGACAGTTACCTTATTCAAACATCAACAACAGGGACCAGATAATTTTTATGGT
GGGACGAGGATACCTGTCTCCAGATCTCAGTAAGGTACGGAGTAACTGTCCAAAAGCCATGAAGAGATTAAT
GGCAGAGTGCCTCAAAAAGAAAAGAGATGAGAGACCACTCTTTCCCCAAATTCTCGCCTCTATTGAGCTGCT
GGCCCGCTCATTGCCAAAAATTCACCGCAGTGCATCAGAACCCTCCTTGAATCGGGCTGGTTTCCAAACAGA
GGATTTTAGTCTATATGCTTGTGCTTCTCCAAAAACACCCATCCAGGCAGGGGGATATGGTGCGTTTCCTGTC
CACTGA-3'

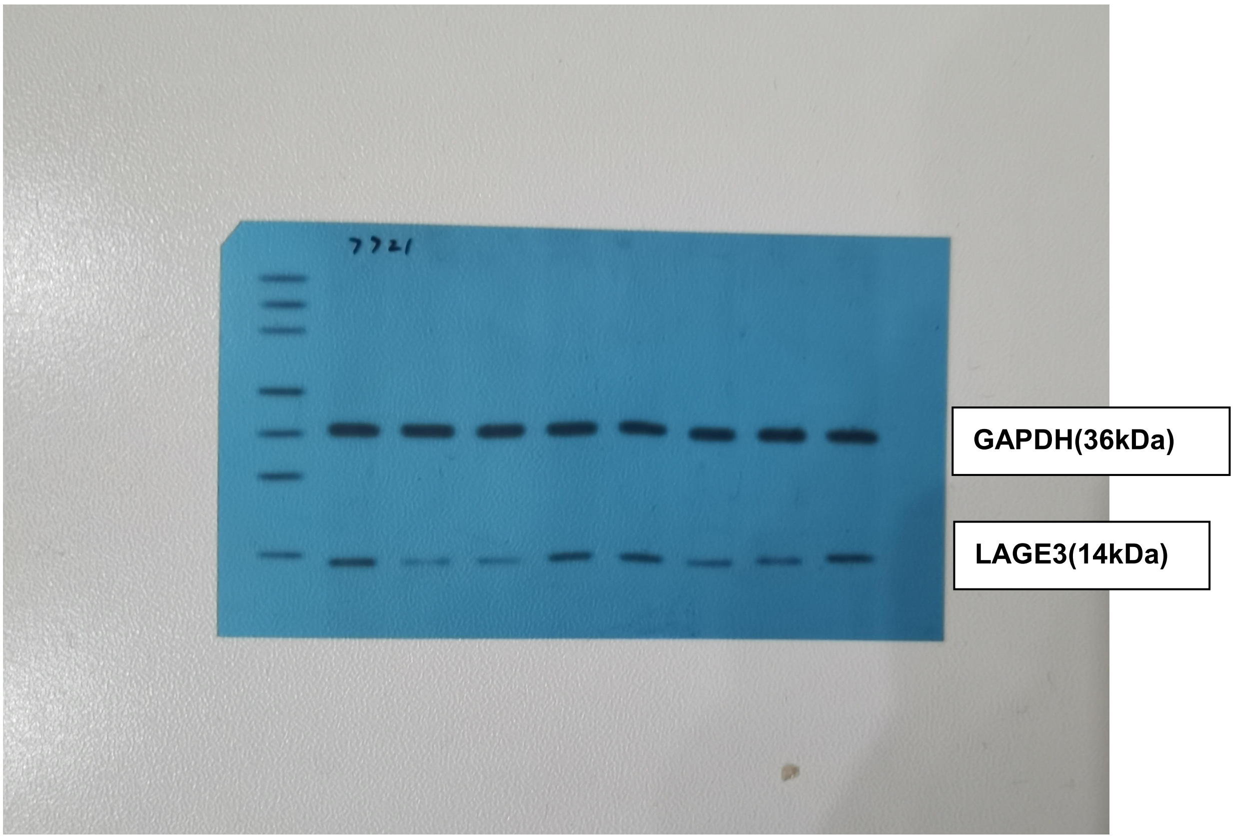

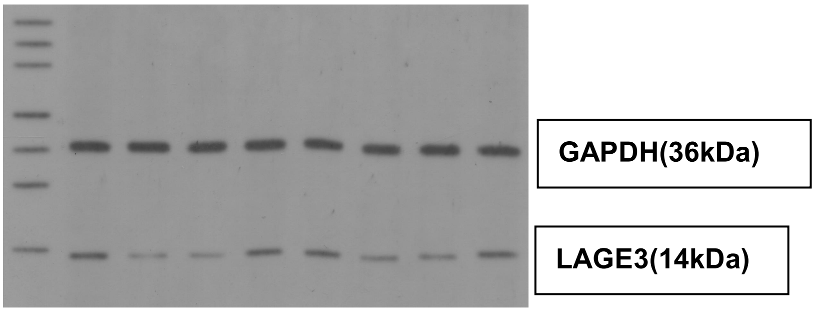

Figure 2C

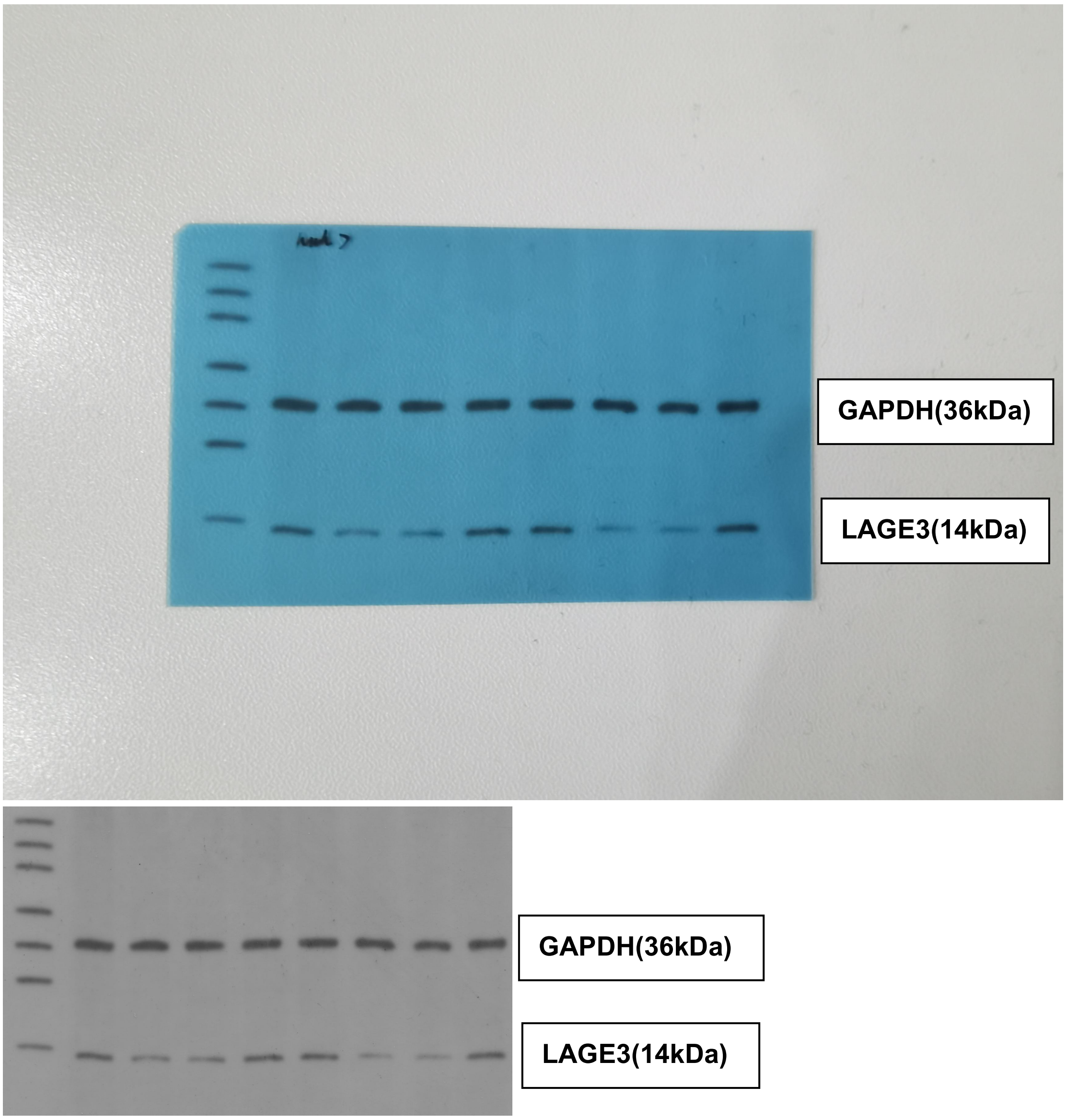

Figure 2D

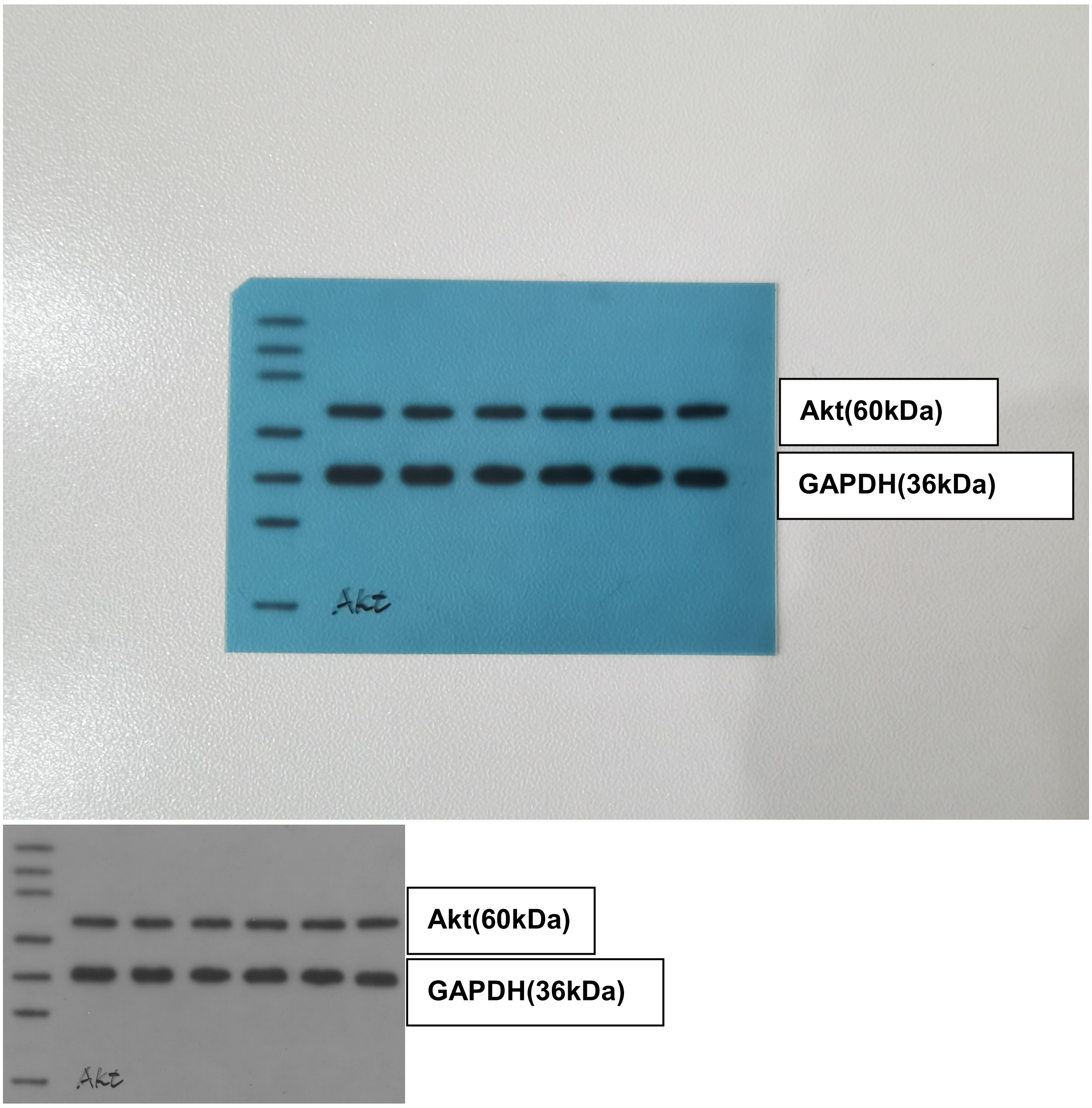

Figure 7B-Akt

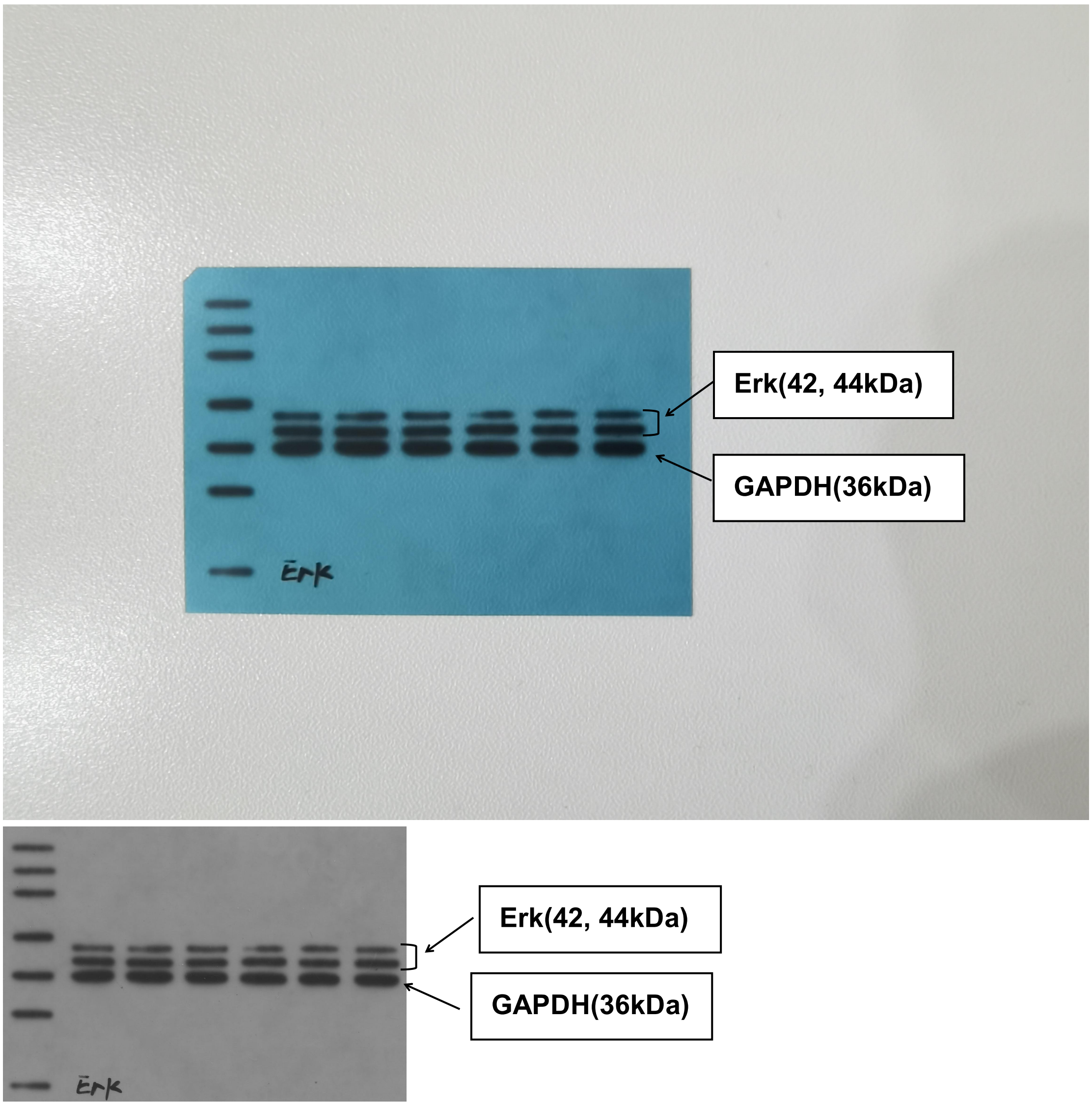

Figure 7B-Erk

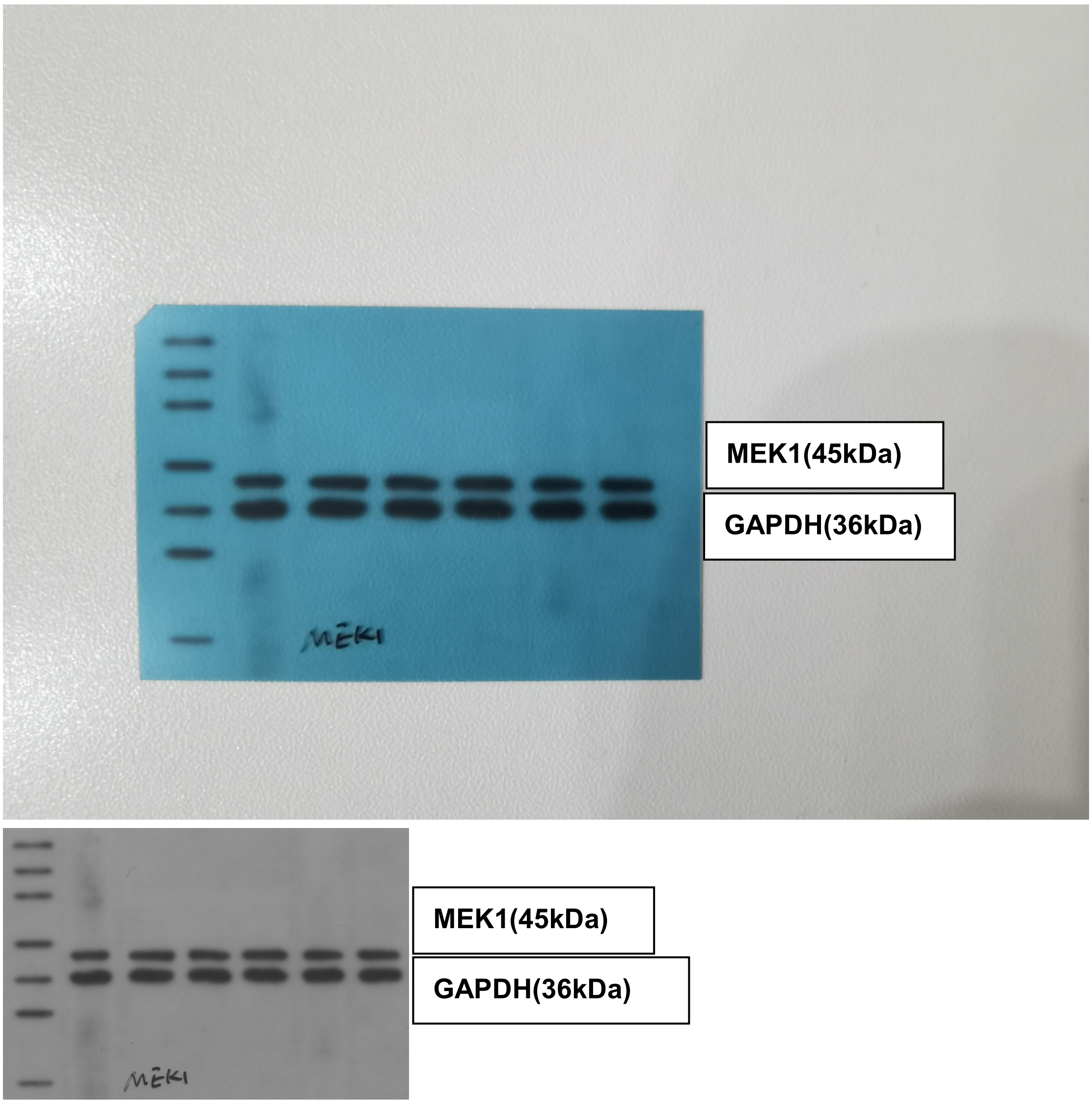

Figure 7B-MEK1

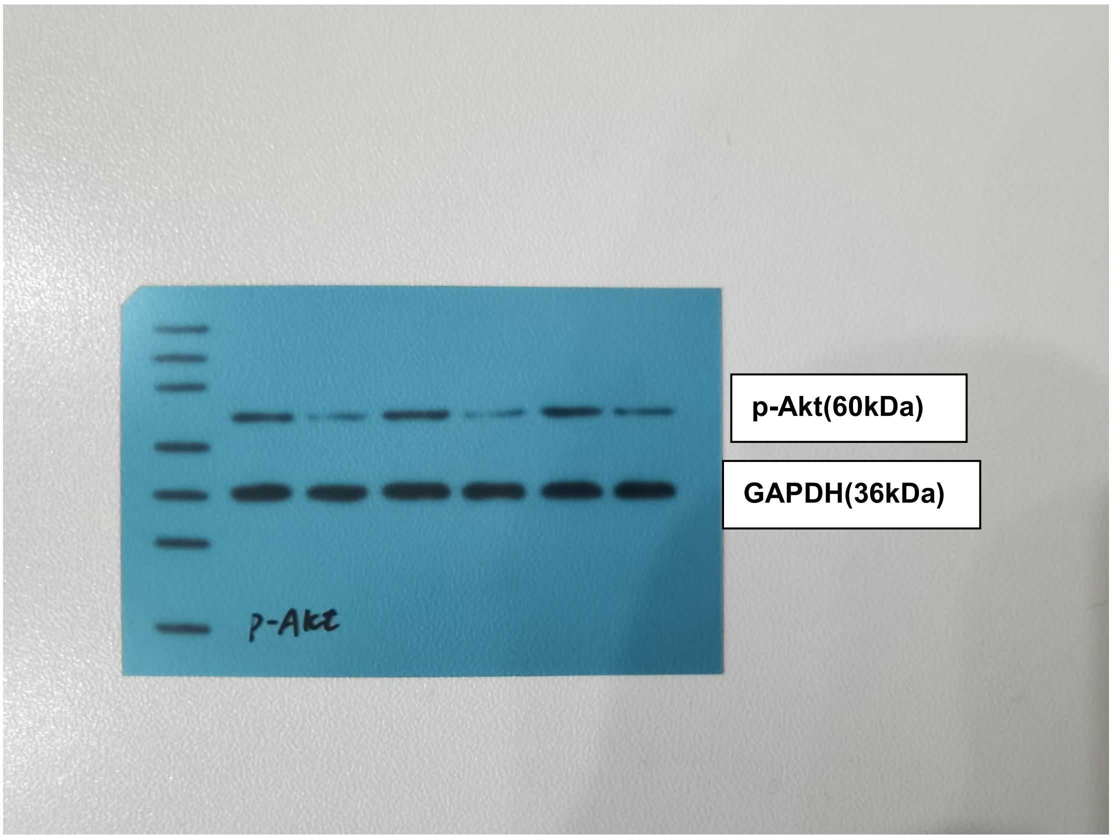

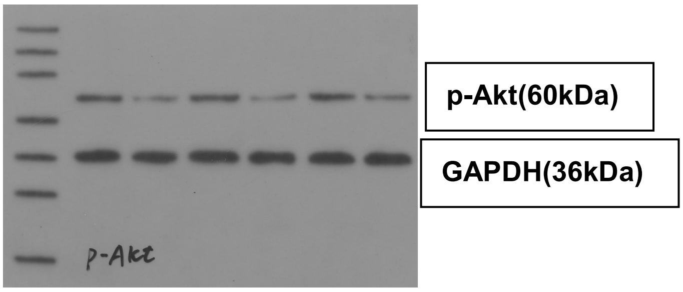

Figure 7B-p-Akt

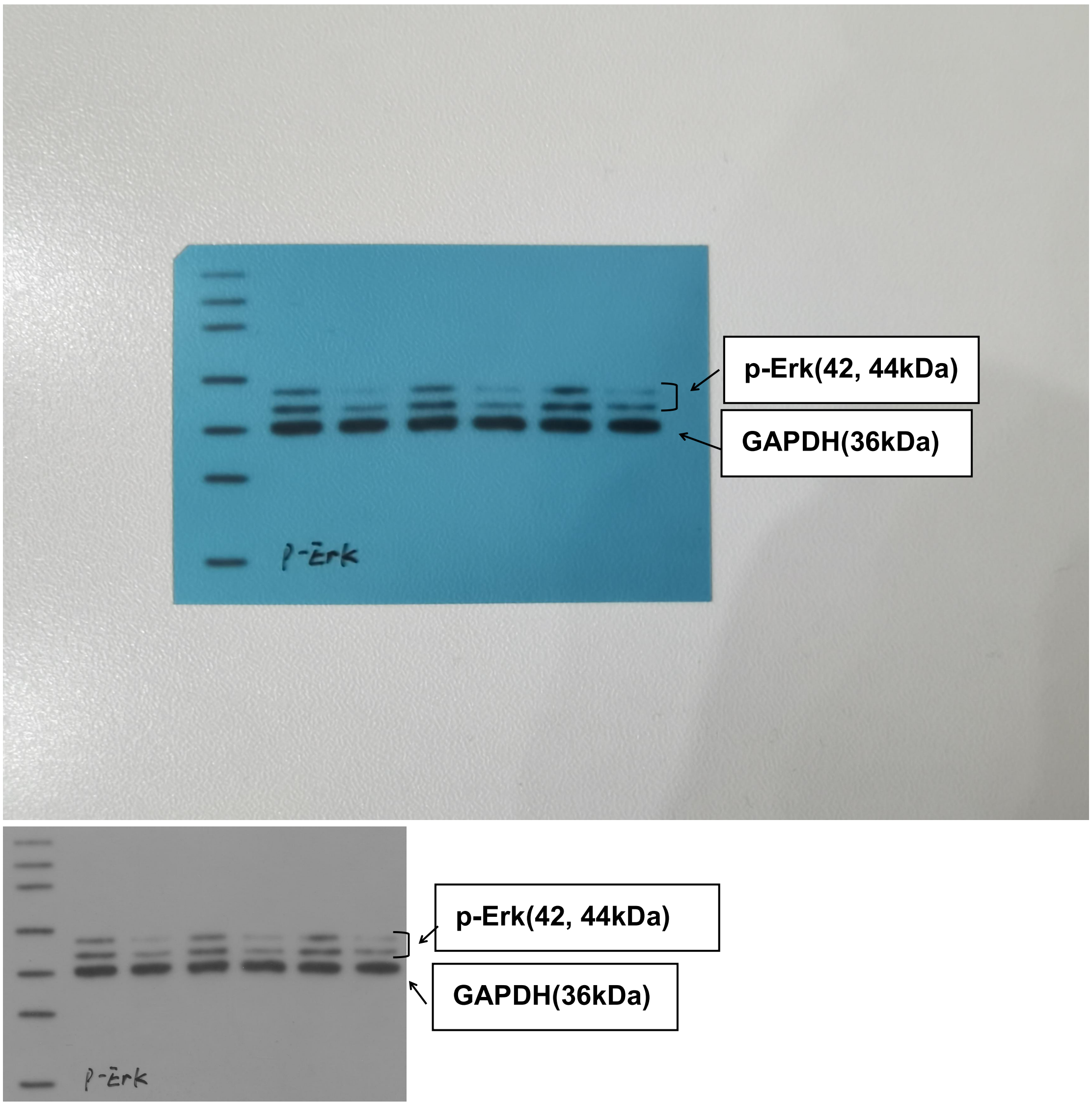

Figure 7B-p-Erk

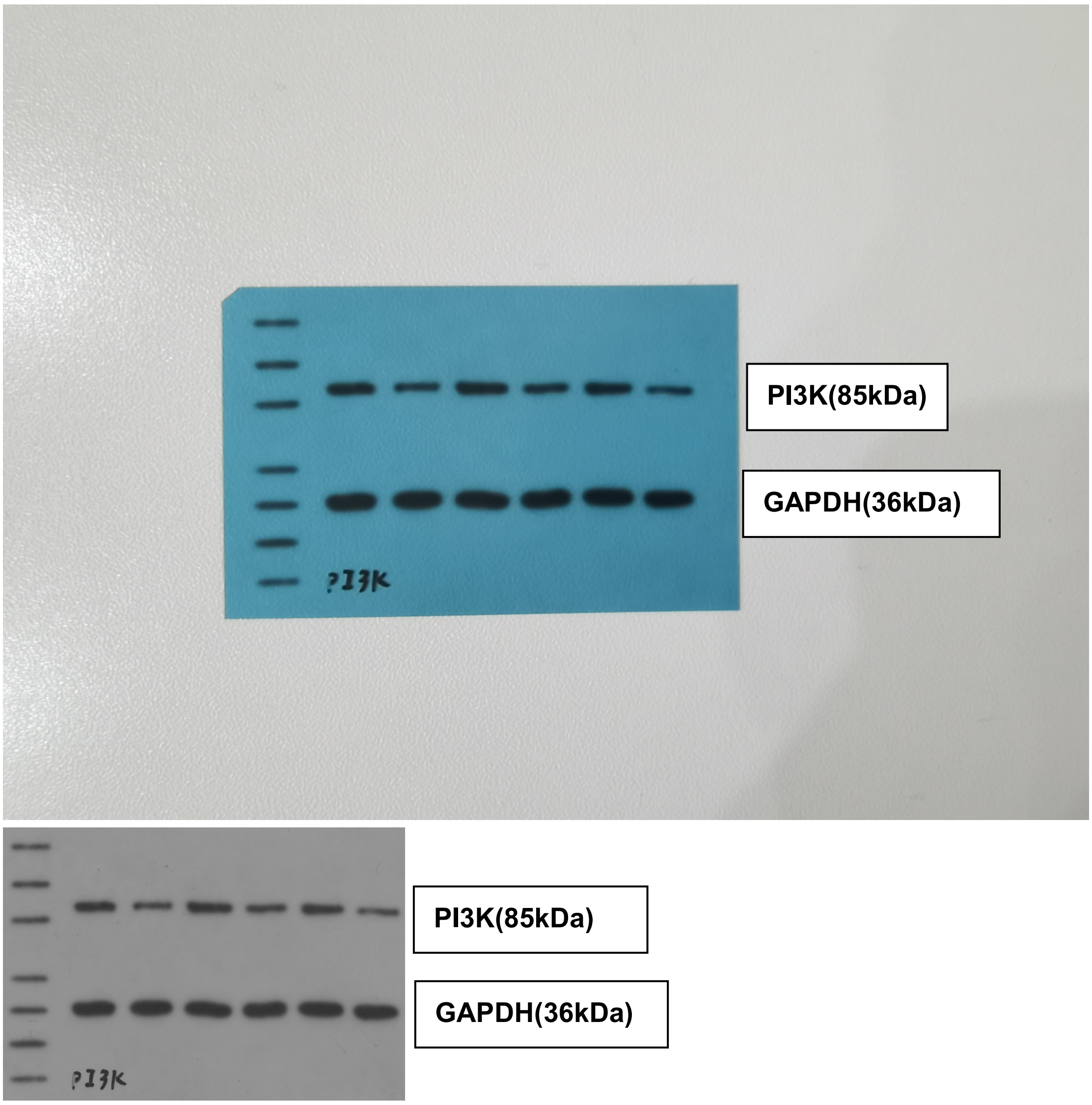

Figure 7B-PI3K

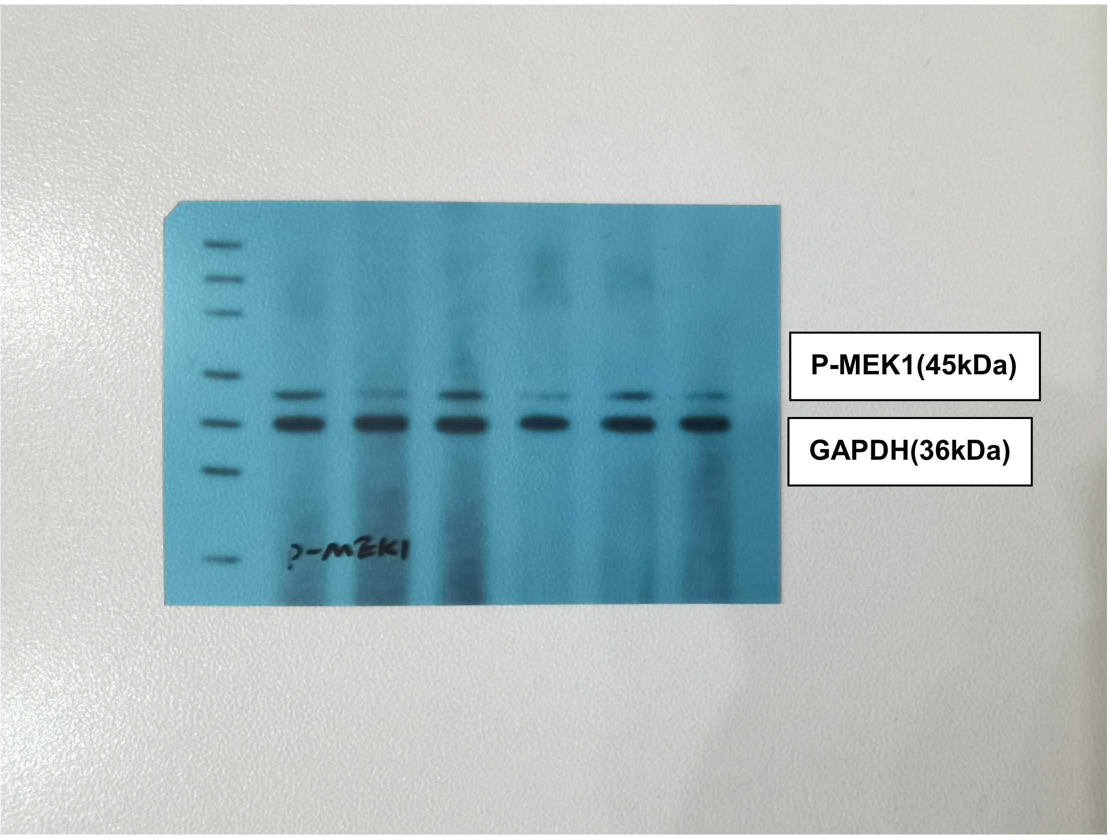

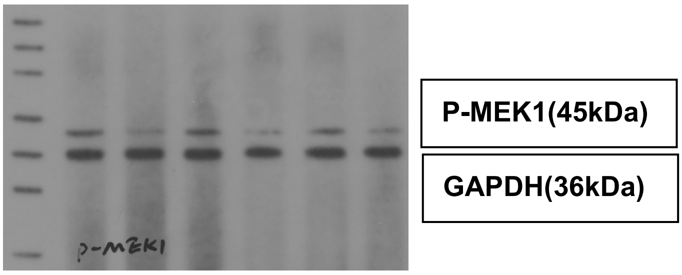

Figure 7B-p-MEK1

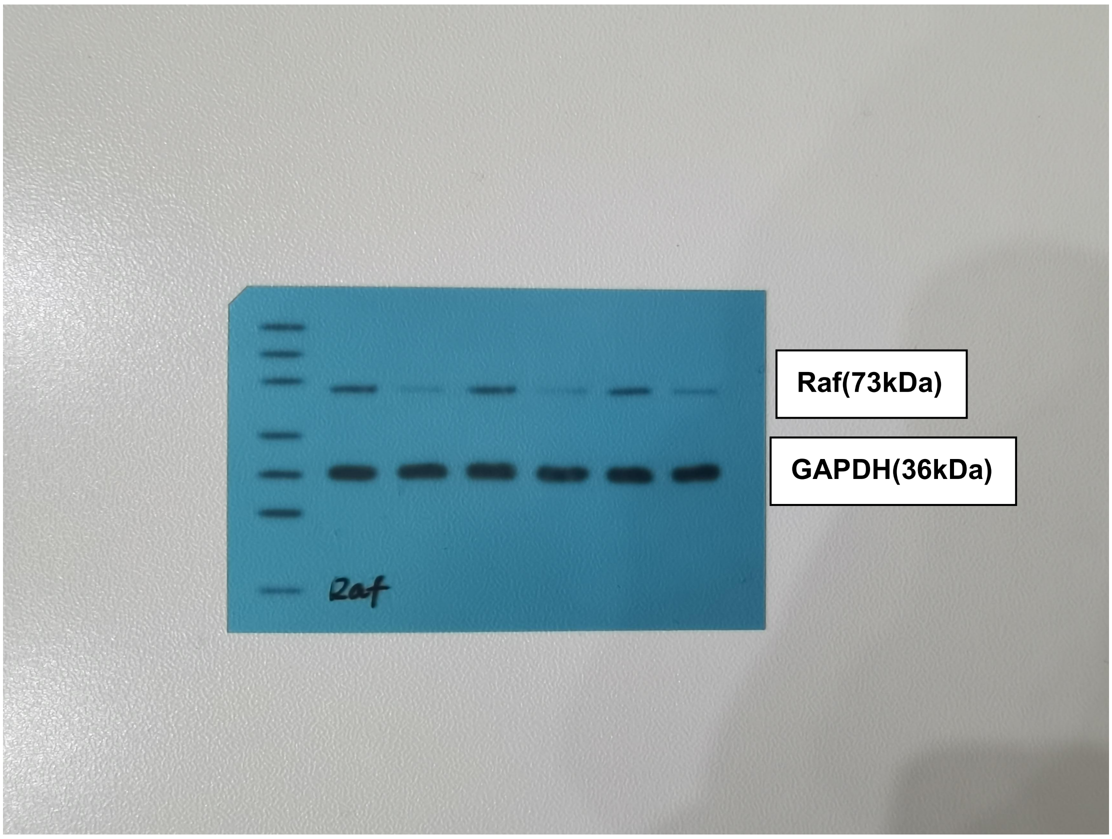

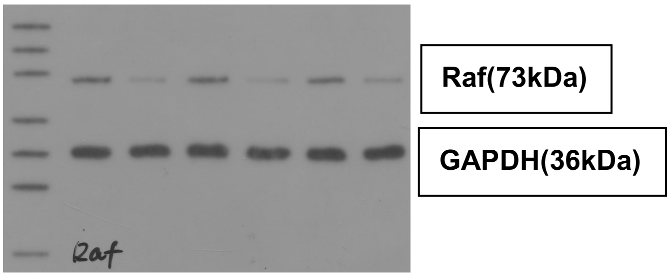

Figure 7B-Raf

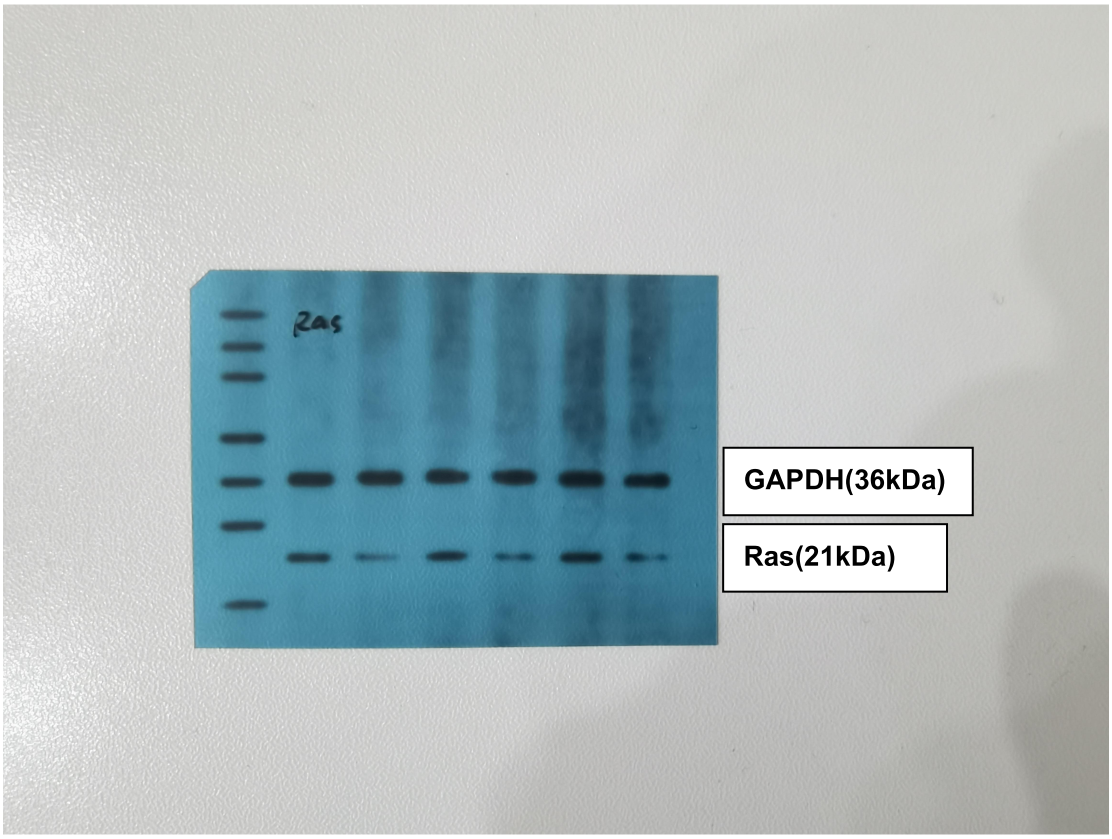

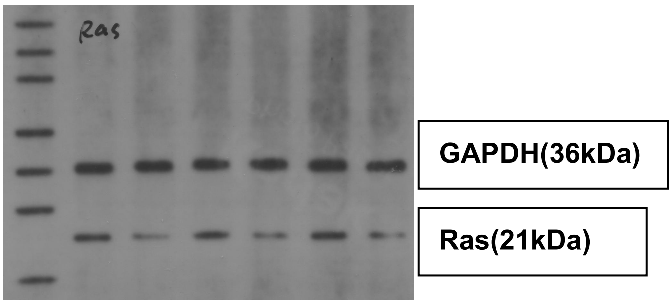

Figure 7B-Ras

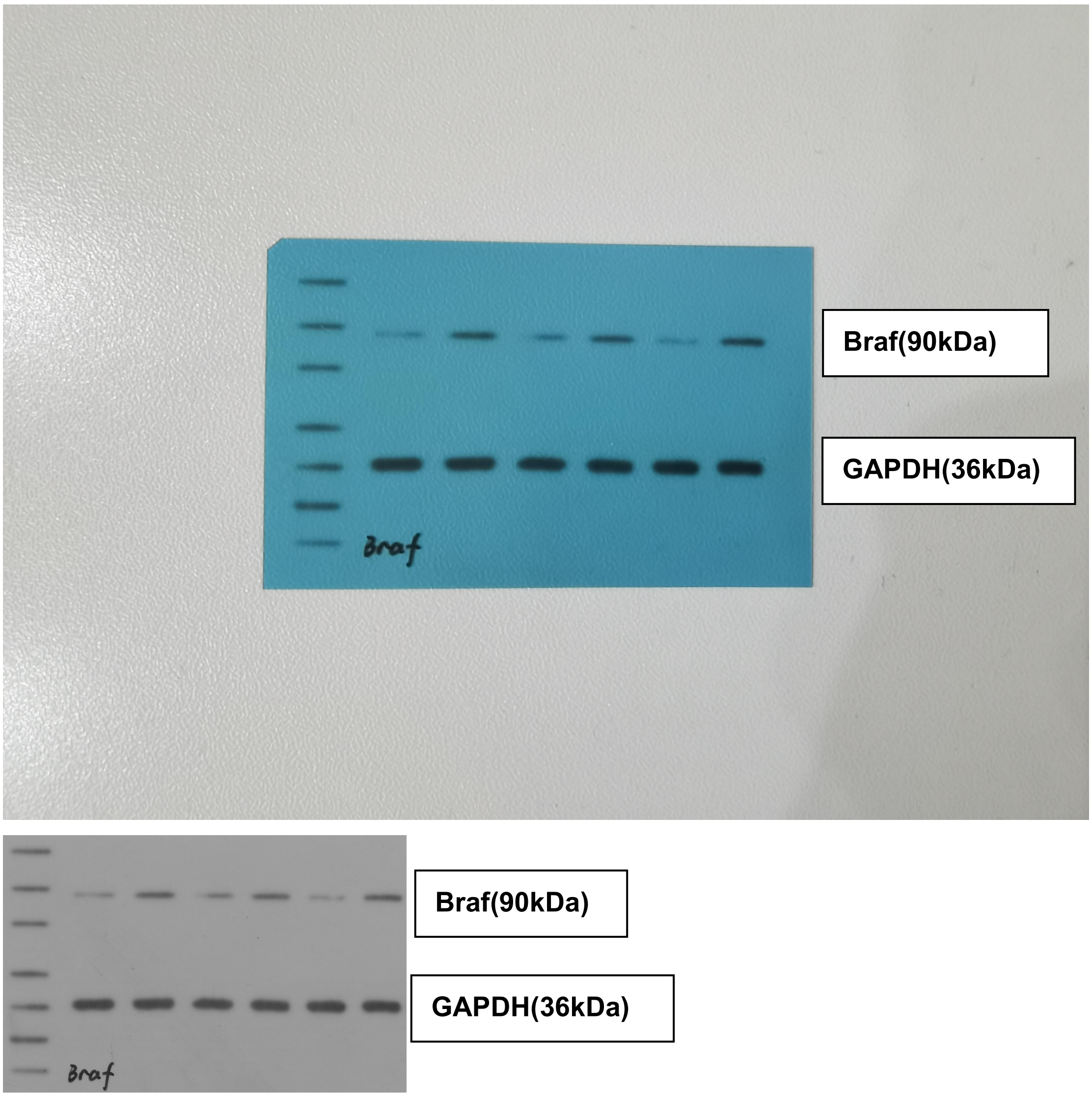

Figure 7C-Braf
